# The dietary management of sodium in children with kidney diseases—clinical practice recommendations from the Pediatric Renal Nutrition Taskforce

**DOI:** 10.1007/s00467-025-06913-z

**Published:** 2025-11-18

**Authors:** José Renken-Terhaerdt, An Desloovere, Michiel JS Oosterveld, Nonnie Polderman, Evelien Snauwaert, Stella Stabouli, Johan Vande Walle, Caroline Anderson, Sheridan Collins, Larry A. Greenbaum, Matthew Harmer, Lyndsay Harshman, Christina L. Nelms, Pearl Pugh, Vanessa Shaw, Jetta Tuokkola, Molly R. Wong Vega, Bradley A. Warady, Rukshana Shroff, Fabio Paglialonga

**Affiliations:** 1 Wilhelmina Children’s Hospital, University Medical Center Utrecht, Utrecht, The Netherlands; 2 Department of Pediatric Nephrology, Ghent University Hospital, Ghent, Belgium; 3 Emma Children’s Hospital, Amsterdam University Medical Center, Amsterdam, The Netherlands; 4 British Columbia Children’s Hospital, Vancouver, BC Canada; 5 Pediatric Nephrology Unit, 1 st Pediatric Department, Hippokration Hospital, Aristotle University of Thessaloniki, Thessaloniki, Greece; 6 University Hospital Southampton NHS Foundation Trust, Southampton, UK; 7 The Children’s Hospital at Westmead, Westmead, Australia; 8 Department of Pediatric Nephrology, Children’s Healthcare of Atlanta, Atlanta, Georgia; 9 Department of Pediatrics, Division of Nephrology, Dialysis and Transplantation, University of Iowa, Iowa City, IA USA; 10 Children’s Mercy Kansas City, Kansas City, MO USA; 11 Nottingham Children’s Hospital, Nottingham University Hospitals NHS Trust, Nottingham, UK; 12 University College London Great Ormond Street Hospital Institute of Child Health, London, UK; 13 Children’s Hospital and Clinical Nutrition Unit, Internal Medicine and Rehabilitation, University of Helsinki and Helsinki University Hospital, Helsinki, Finland; 14 Texas Children’s Hospital, Houston, TX USA; 15 Department of Pediatric Nephrology, Children’s Mercy Kansas City, Kansas City, MO USA; 16 Pediatric Nephrology, Dialysis and Transplant Unit, Fondazione IRCCS Ca’ Granda Ospedale Maggiore Policlinico, Via Commenda 9, Milan, 20122 Italy

**Keywords:** Sodium, Salt, Kidney diseases, Pediatric Renal Nutrition Taskforce, Clinical practice recommendations

## Abstract

Sodium imbalance is a common concern in children with kidney diseases, presenting as either sodium excess or sodium deficit, each with significant clinical implications. Sodium excess contributes to fluid overload and hypertension, while increased sodium losses, particularly via urine or peritoneal fluid, can predispose patients to hypotension and growth failure. Effective sodium management is thus a critical component of care in pediatric kidney diseases, with dietary sodium intake playing a pivotal role in maintaining homeostasis. The Pediatric Renal Nutrition Taskforce (PRNT) has developed clinical practice recommendations (CPRs) for dietary sodium management in children with kidney diseases, addressing key aspects of sodium balance, including primary dietary sources, nutritional assessment of sodium intake, non-dietary factors influencing sodium balance, and recommended intakes. Strategies for reducing sodium intake in cases of sodium excess and augmenting intake in patients with increased sodium losses are also provided. The consensus of international experts was assessed through a Delphi process. These CPRs will be regularly updated by the PRNT.

**Supplementary Information:**

The online version contains supplementary material available at 10.1007/s00467-025-06913-z.

## Introduction

The kidneys play a crucial role in regulating sodium homeostasis, which accounts for the increased susceptibility of children with kidney diseases to sodium overload or depletion. Sodium excess can result in fluid retention and hypertension, the main modifiable cardiovascular risk factors for patients with kidney diseases. Alternatively, sodium depletion can result from excessive urinary sodium excretion, as observed in children with kidney dysplasia or tubulointerstitial disease, or from uncompensated sodium losses in the dialysis fluid, particularly in infants undergoing peritoneal dialysis (PD). Sodium depletion may contribute to growth failure and hypotension.

Dietary sodium intake is the primary determinant of sodium balance. Observational studies in healthy children and adults with chronic kidney disease (CKD) demonstrated a strong causal association between dietary sodium intake and blood pressure (BP) [1–3]. Nevertheless, the paucity of well-designed trials in children with kidney diseases and the lack of evidence-based clinical practice guidelines present a significant challenge in effectively addressing this issue in clinical practice. Notably, children with kidney diseases consume, on average, more sodium than recommended for healthy children [4].

Therefore, PRNT has developed clinical practice recommendations (CPRs) to guide clinicians in the management of dietary sodium intake in children with kidney diseases. Key evidence points and clinical practice points were provided to describe the dietary sources of sodium, assessment of dietary sodium intake, non-dietary factors impacting sodium balance, and to optimize dietary sodium intake.

### Terminology

Table 1 provides definitions of terms pertaining to sodium. In the current CPRs, the term “sodium” is preferentially used, whereas the term “salt” is commonly employed in clinical practice to describe the sodium content in food and is often used interchangeably with “sodium chloride.” Salt (sodium chloride) comprises 40% sodium and 60% chloride; the amount of sodium in grams can be determined by multiplying the amount of salt in grams by 0.394.

**Table 1:** Definition of terms concerning sodium and salt

Term	Definition	
Sodium	Chemical element with atomic number 11 and indicated by the symbol Na (for Natrium) Molecular weight 23 Da (1 mmol = 23 mg)	
Salt (sodium chloride)	Ionic compound consisting of sodium and chlorine ions and with the chemical formula NaCl Molecular weight 58 Da (1 mmol = 58 mg) 1 g NaCl contains 0.4 g of Na^+^ 2.5 g NaCl contains 1 g of Na^+^ (grams of sodium = grams of salt × 0.394)	
Naturally occurring sodium/salt	Intrinsic or inherent sodium/salt	
Added sodium/salt	Sodium/salt added during the cooking process, at the table, or in (ultra) processed food	
Reduced sodium/salt	Products with a sodium content that is reduced by at least 25% compared to that of similar products.^1,2^	
Fortified table salt	Commercially available salt to be used as “added salt” as defined above. Contains micronutrients such as iodine in sufficient amounts to improve the nutritional quality.^3,4^	
Iodized salt	Table salt mixed with minute amounts of iodinated salts (usually potassium iodate or potassium iodide); the amount of sodium shall be established by respective national health authorities.^5^	
Low-sodium salt substitute (LSSS)	Products with less sodium than regular salt. Amounts of sodium in LSSS are lowered by replacing some of the sodium with potassium or other minerals. LSSS is marketed to reduce the risk of high blood pressure and cardiovascular disease associated with a high intake of sodium chloride while maintaining a similar taste. The most used salt substitute is potassium chloride.^6^	
Processed food	Products made by adding salt, oil, sugar, and using preservation methods such as canning and bottling, and, in the case of breads and cheeses, using non-alcoholic fermentation.^7^	
Ultra-processed food	Formulations of ingredients, mostly of exclusive industrial use, made by a series of industrial processes, many requiring sophisticated equipment and technology.^7^	
DEFINITION OF FOODS ACCORDING TO SODIUM CONTENT		
	USA and Canada^8^	Europe^9^
Sodium/salt free	Less than 5 mg of sodium per serving.	The product contains no more than 5 mg of sodium, or the equivalent value for salt, per 100 g.
Very low sodium/salt	35 mg of sodium or less per serving.	The product contains no more than 40 mg of sodium, or the equivalent value for salt, per 100 g or per 100 ml. Excluded are mineral waters and other waters.
Low sodium/salt	140 mg of sodium or less per serving.	The product contains no more than 120 mg of sodium, or the equivalent value for salt, per 100 g or per 100 ml. For waters, other than natural mineral waters falling within the scope of Directive 80/777/EEC, this value should not exceed 2 mg of sodium per 100 ml.
Lower in sodium/salt/reduces sodium/salt	At least 25% less sodium than the regular product.	n.a.
Light in sodium/lightly salted	At least 50% less sodium than the regular product.	n.a.
No added sodium/salt/unsalted	No salt is added during processing – but these products may not be salt/sodium-free unless stated.	The product does not contain any added sodium/salt or any other ingredient containing added sodium/salt and the product contains no more than 120 mg sodium, or the equivalent value for salt, per 100 g or 100 ml.

References

^1^https://www.fda.gov/media/84261/download

^2^ European Commission; Knowledge for policy; Health promotion and disease Prevention Knowledge Gateway. https://knowledge4policy.ec.europa.eu/health-promotion-knowledge-gateway/dietary-saltsodium_en#:~:text=References-,Defining%20dietary%20salt%20and%20sodium,and%200.6%20g%20of%20chloride. Last updated 26 oct 2023 Assessed Febr 2024

^3^ WHO food fortification https://www.who.int/health-topics/food-fortification#tab=tab_1 Assessed Febr 2024

^4^NAFDAC 2019 https://extranet.who.int/nutrition/gina/sites/default/filesstore/NGA_2019_Food-Grade-Table-Cooking-Salt-Regulations-2019.pdf Assessed Febr 2024

^5^ CODEX STAN 150-190 https://www.fao.org/fao-who-codexalimentarius/sh-proxy/en/?lnk=1&url=https%253A%252F%252Fworkspace.fao.org%252Fsites%252Fcodex%252FStandards%252FCXS%2B150-1985%252FCXS_150e.pdf Assessed Febr 2024

^6^Brand A, Visser ME, Schoonees A, Naude CE. Replacing salt with low-sodium salt substitutes (LSSS) for cardiovascular health in adults, children and pregnant women. Cochrane Database Syst Rev. 2022 Aug 10;8(8):CD015207. doi: 10.1002/14651858.CD015207. PMID: 35944931; PMCID: PMC9363242.

^7^ Crimarco A, Landry MJ, Gardner CD. Ultra-processed Foods, Weight Gain, and Co-morbidity Risk. Curr Obes Rep. 2022 Sep;11(3):80-92. doi: 10.1007/s13679-021-00460-y. Epub 2021 Oct 22. PMID: 34677812; PMCID: PMC8532572.European Commission; Food safety. Labelling and Nutrition.

^8^https://www.canada.ca/content/dam/hc-sc/documents/services/technical-documents-labelling-requirements/table-permitted-nutrient-content-statements-claims/table-document/table-of-permitted-nutrient-content-statements-and-claims.pdf

^9^ Regulation (EC) No 1924/2006 of the European Parliament and of the Council of 20 December 2006 on nutrition and health claims made on foods. https://eur-lex.europa.eu/legal-content/EN/TXT/PDF/?uri=CELEX:32006R1924&qid=1731676425372

Various terms are utilized in literature to distinguish different sources of salt. Naturally occurring salt (also referred to as intrinsic salt) denotes the sodium chloride inherently found in food. “Added salt” refers to salt that is added during food preparation, at the table, or in different forms, in the manufacture of processed foods (e.g., sodium chloride, monosodium gluconate, disodium phosphate, sodium bicarbonate). The terms “visible salt” and “hidden salt” are not included in these CPRs due to their potential for misinterpretation.

## Methods

The PRNT composition, the CPRs development process, grading of evidence, and plans for audit and revision of the CPRs have been described in earlier work from the PRNT [5]. In summary, evidence review and CPR development were performed by the core group of PRNT members and reviewed through a Delphi consensus process by a voting panel.

### The PICO questions

The PICO (Patient, Intervention, Comparator, and Outcome) format was used within the CPR to address questions. The PICO terms were as follows:

- Population: children from birth to 18 years of age with kidney diseases. Children with acute kidney injury, primary tubulopathies, nephrolithiasis, and nephrocalcinosis were not considered in this document as they require distinct sodium management plans.
- Intervention: recommended sodium intake from diet and therapy
- Comparator: recommended sodium intake in age-matched healthy children or no comparator
- Outcomes: BP and volume status; growth in infants and children less than 2 years old.

As outcomes, we focused on BP and fluid status, as these are recognized as the main endpoints associated with sodium intake. We included growth as an outcome in infants and children until 2 years of age, given that growth failure is a known consequence of chronic sodium and fluid depletion in this population. Urinary sodium is not a reliable outcome measure in people with kidney diseases and was not used.

### Literature search

Publications on PubMed, Medline, Embase, and the Cochrane Library from 2000 to 2024 were searched. Publications on dietary sodium sources, intake, and effects in children with kidney diseases were reviewed and used to provide key evidence points and clinical practice points for this population. Details of the literature search criteria are described in Supplementary Table 1. Given the lack of randomized controlled trials (RCTs) on the requirements or effects of sodium intake in children with kidney diseases, observational studies (irrespective of number of patients) and retrospective studies with more than 20 patients were included. Also, RCTs and meta-analyses focusing on sodium intake in healthy children, and meta-analyses of RCTs in adults with kidney diseases have been considered, and where appropriate, extrapolated to children with kidney diseases. Where suitable studies were lacking, the opinion of experts from the PRNT was utilized, but must be carefully considered by clinicians and adapted to individual patient needs as appropriate. Appendix A has an extended list of papers pertaining to the topics discussed in these CPRs.

### Framing advice

Statements were graded in accordance with the American Academy of Pediatrics recommendations (Supplementary Fig. 1) [6] and submitted to a Delphi procedure to validate expert opinion, as elsewhere described [5].

## Clinical practice recommendations

**Q1. What are the main dietary sources of sodium for an infant, child, and adolescent?**

## Key evidence points

1.1 Sodium intake varies across regions and between countries due to cultural differences in consumption, cooking practices, taste preferences, and differences in food-processing methods (ungraded).
1.2 The major sources of sodium are processed and ultra-processed foods through the use of added salt and sodium-containing additives (grade B, moderate).

## Rationale

### Healthy children

Studies investigating sodium sources often yield non-comparable results due to variations in the classification of food groups. A particular food category may be identified as a major sodium source based on either its high consumption volume (e.g., bread) or its inherently high sodium content (e.g., processed luncheon meats and soups).

Cultural variations in consumption patterns, cooking practices, taste preferences, and food-processing methods across countries contribute to differences in sodium intake [7].

Sodium intake originates from three primary sources: naturally occurring sodium in foods, added salt, and pharmaceutical products. In most cultures and regions, naturally occurring sodium accounts for a minor proportion of total sodium intake. Examples include raw meat and fish (30–150 mg/100 g), fruits and vegetables (< 50 mg/100 g), tap water (depending on the source, 9.5 mg/L [range: 4.3–20.0 mg/L]), and bottled mineral water (1–1419 mg/L in Europe) [8].

**Fig. 1:**
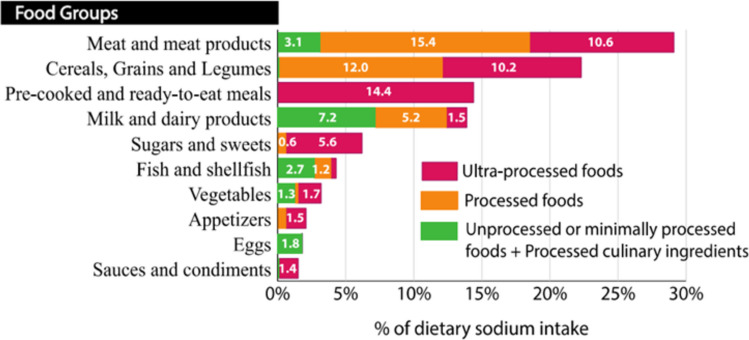
Main contributors to dietary sodium intake from ultra-processed, processed, and unprocessed foods in children (from Crimarco, 2022) Contribution of the sodium content in different food groups to the total sodium dietary intake among children in Spain. Foods were classified based on the degree of processing according to the NOVA system They were organized into three groups in this figure: unprocessed or minimally processed foods + processed culinary ingredients, processed foods, and ultra-processed foods [11]

**Fig. 2:**
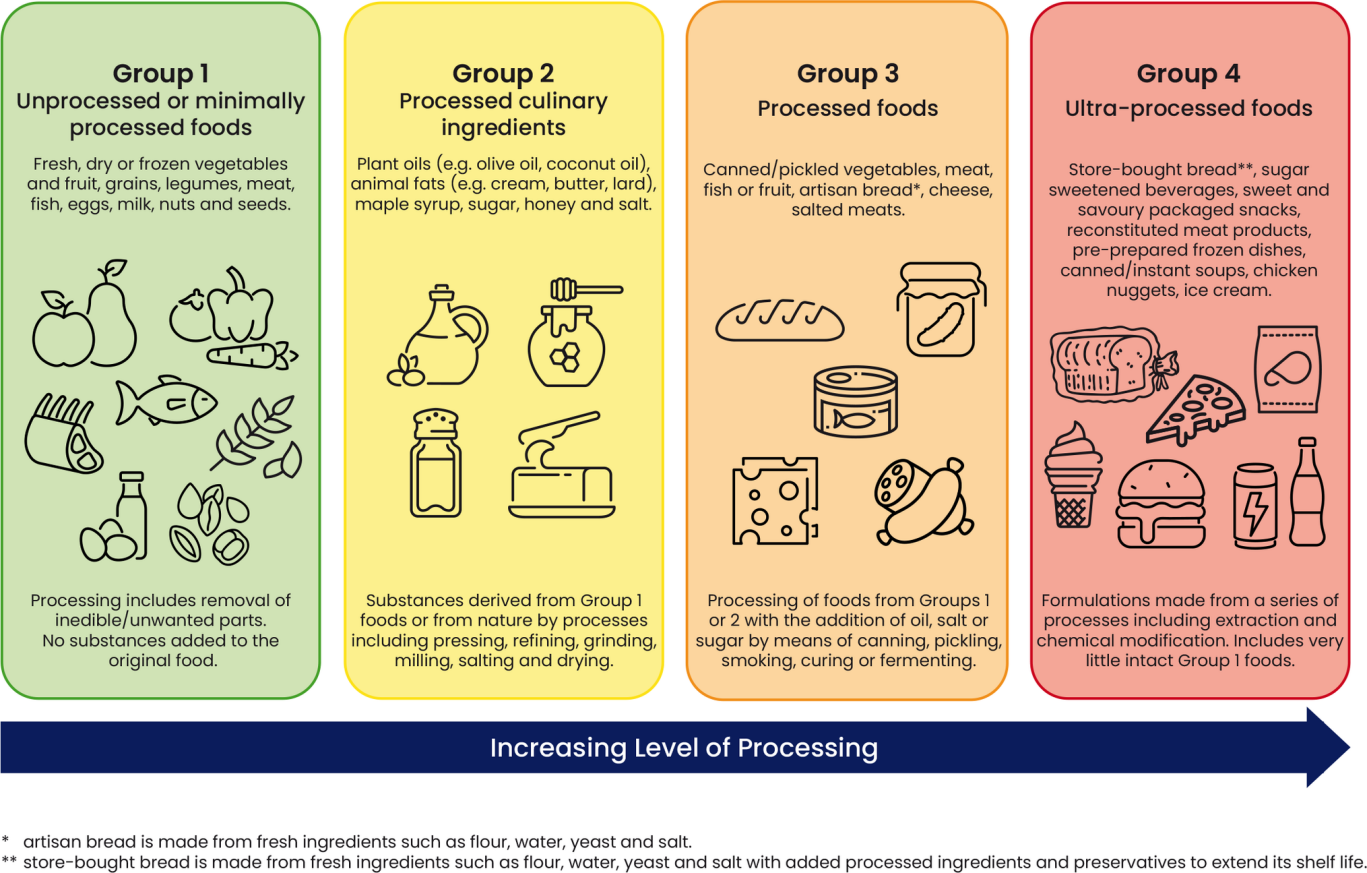
Classification of foods according to level of processing, adapted from Crimarco 2022 [11]

Salt is commonly added for food processing and preservation, during home preparation, and at the table [8, 9]. Sodium intake can increase by as much as 60% when consuming a diet with additives (see Fig. 1) [10]. Not all countries require additives to be listed on labels. If listed, sodium-containing additives can be recognised by the presence of sodium in the name. In the European Union, the E-numbers of the additive may also be used (see Supplementary Table 2). Foods may be classified by their level of processing and additive content according to the NOVA classification system (see Figs. 1 and 2) [11]. Processed foods, which include not only fast foods and convenience items but also Bread, Breakfast cereals, and cheese and pre-prepared meat, fish, and vegetables, are the predominant contributors to sodium intake in Western diets, accounting for 77 to 83% of total sodium intake [12–14]. In the USA, the top 15 food categories contributing to dietary sodium are consistent across population subgroups defined by age, sex, race, and household income; leading contributors include pizza (5.3%), bread, rolls, and buns (4.7%), cold cuts and cured meats (4.6%), soups (4.4%), burritos and tacos (4.3%), and savory snacks (4.1%) [15]. In European populations, the main contributors include bread, meat and meat products, and cheese and dairy products [7].

The contribution of salt added during food preparation or at the table is relatively minor when compared to sodium added during food processing; in the United Kingdom, 5.6% of sodium intake is attributed to preparation, and 4.9% to table salt. Similar trends are observed in other European countries [9], while in Canada the combined contribution is 11% [12, 13].

Analyses of sodium intake at different times of the day reveal that the highest sodium consumption occurs during dinner (36–39%), followed by lunch (30–31%), snacks (16–19%), and breakfast (14–15%) [16].

Individuals with lower socioeconomic status tend to consume greater amounts of high-sodium processed foods [7]. In many populations, adolescents tend to consume significantly more meat, packaged sweets, and snacks than recommended, suggesting a higher proportion of their sodium intake originates from such sources compared to other demographic groups [7].

### Children with kidney disease

The Chronic Kidney Disease in Children (CKiD) study, an ongoing multicentre prospective cohort study of children with mild-to-moderate CKD, found that fast foods provided 8.8% and 10.2% of daily sodium intake in the 6–13 and 14–18 year age groups, respectively. Cheese accounted for 10% of daily sodium intake in the 2–5 year age group. No significant differences in sodium intake were observed across different CKD stages [4]. Although limited to a single study, these data suggest that processed and ultra-processed foods are a major source of sodium intake in children with CKD.

**Q2. How is dietary sodium intake assessed in children?**

## Clinical practice points

2.1 We suggest using a diet recall of a typical 24-h period or a food frequency questionnaire to identify the main dietary sources of sodium (grade C, weak).
2.2 We suggest using a 3-day prospective diet diary for an in-depth analysis of sodium intake (grade C, weak).
2.3 We do not recommend the routine measurement of serum sodium or urinary sodium to estimate the sodium intake or sodium balance in children with kidney diseases (grade C, weak).

## Rationale

### Dietary sodium intake assessment

A retrospective diet recall of a typical 24-h period can identify the main dietary sources of sodium. Food frequency questionnaires (FFQs), tailored to focus on the consumption of high-sodium foods, may also be helpful for collecting information on eating patterns. For more comprehensive data collections, a 3-day prospective diet diary or food intake record may be used.

Quantifying the exact amount of sodium consumed is not possible unless the infant or child is exclusively fed with a formula or by enteral tube feed. Limitations of the assessment tools to determine dietary sodium intake are well documented and include difficulties in accurately measuring added salt (both during cooking and at the table), estimating portion sizes, and inaccuracies in food composition databases [17].

Several systematic reviews in the adult population examined dietary sodium assessment methods and their comparison with urinary sodium excretion, which was used as the gold standard method in large trials [18–20]. McLean studied the reliability and validity of FFQs for estimating dietary sodium intake by reviewing the findings from 18 studies. Significant variability in study design and analysis was reported, and poor agreement between FFQs and 24-h urinary sodium excretion [18]. Subsequent investigations showed that 24-h diet recalls and diet records inaccurately estimate dietary sodium intake when compared to 24-h urinary excretion [19]. More recently, McLean confirmed that 24-h diet recalls tend to underestimate sodium intake, although accuracy can be improved with a “high quality 24-h diet recall,” which includes accurate food composition databases, measurement of discretionary salt and the multiple-pass method: this consists in a 5-step interview that includes reference to frequently forgotten foods as one of the stages of review [20].

### Urinary and serum sodium

The use of urinary sodium to estimate sodium intake in healthy adults is highly controversial. A single complete 24-h urine collection is considered to provide a reliable estimate of the average current dietary sodium intake at a population level, but not of individual sodium intake [21, 22]. A significant day-to-day variability in 24-h urinary sodium has been reported, even in individuals maintained on a fixed sodium diet for months [23], mainly due to the presence of stores of non-osmotically active sodium in various body tissues, notably the skin [24]. At least three non-consecutive 24-h urine collections are recommended to provide a reasonably accurate estimate of an individual’s sodium intake in healthy adults [22].

Some formulas have been proposed to estimate sodium intake from spot collections, but they tend to overestimate sodium intake at lower 24-h urine sodium levels and underestimate intake at higher 24-h urine sodium. A trial in adults with CKD confirmed that these formulas have low precision and accuracy [25]. They are currently not recommended for assessing an individual’s sodium intake [22].

The limitations of urinary sodium to estimate sodium intake may be more pronounced in individuals with kidney diseases that affect urinary sodium excretion, either by reducing filtration or tubular reabsorption. In addition, obtaining complete 24-h urine collections can be challenging in pediatrics. Considering these limitations and given the lack of specific trials in children with kidney diseases, caution should be exercised when using urinary sodium to estimate sodium intake in this population.

Although both hyponatremia and hypernatremia are associated with higher risk of all-cause mortality in adults with CKD, serum sodium cannot be used as a measure of sodium intake or balance, as it is influenced by many other factors. Imbalances in serum sodium concentration are primarily caused in CKD by the inability of the kidneys to regulate fluid homeostasis, with volume overload being a common cause of hyponatremia in this population.

**Q3. What are the non-dietary factors contributing to sodium balance?**

## Key evidence points

3.1 Medications can be a significant source of sodium (ungraded).
3.2 Dialysis can lead to a gain or loss of sodium depending on the individual’s sodium balance and the dialysis prescription (grade C, moderate).
3.3 Salt-wasting kidney diseases and extra-renal losses may lead to sodium depletion in infants and young children (grade C, moderate).

## Rationale

### Medications

As shown in Table 2, some drugs, including many over-the-counter effervescent or soluble medications, contain a significant amount of sodium and are associated with a higher risk of hypertension and cardiovascular events, the latter in both hypertensive and non-hypertensive adults [26]. This is particularly important in patients with CKD. For example, sodium-based potassium exchange resins are associated with higher interdialytic weight gain (IDWG) in adults receiving maintenance hemodialysis (HD) [27]. Formula decanted with these drugs increased sodium content by 86–260% [28]. A recent prospective observation study of 41 children on dialysis showed that 30% received sodium-containing drugs, which accounted for around 40% of the total sodium intake [29]. Notably, some of these drugs can be replaced with non-sodium-containing alternatives (e.g., calcium-based instead of sodium-based potassium exchange resins), offering an important strategy in hypertensive patients with evidence of sodium and fluid overload.

**Table 2:** Examples of sodium content of some commonly used medications

Drug	Dose	Sodium content (mmol/dose)	Sodium content (mg/dose)
**Oral drugs**			
**Frequently used in patients with kidney disease**			
Polystyrene sulfonate oral powder	15 g dose	60	1380
Sodium zirconium cyclosilicate	5 g sachet	17.4	400
Sodium bicarbonate 500 mg capsules	1 capsule	6	138
Sodium alginate + sodium bicarbonate, 500 + 267 mg tablets	1 tablet	2.6	60
Sodium chloride 1 mmol/ml solution	1 ml	1	23
**Common over the counter medications**			
Ibuprofen 600 mg effervescent granules	1 sachet	8.6	198
Paracetamol 500 mg effervescent tablets	1 tablet	18.2–19	419–437
Paracetamol 500 mg soluble tablets	1 tablet	16.9–17	389–391
Cold and Flu Relief powder	1 sachet	5.1	117
Acetylcysteine 600 mg effervescent tablets	1 tablet	15.5	356
Calcium gluconate 1 g effervescent tablets	1 tablet	4.5	103
Movicol 13.8 g oral powder	1 sachet	8.1	186
Oral rehydration solutions	100 ml	6–9	138–207
**Intravenous drugs**			
Normal saline (0.9% NaCl)	1 L	154	3500
Half saline (0.45% NaCl)	1 L	77	1750
Ringer’s lactate	1 L	132	3036
Sodium bicarbonate 8.4%	1 ml	1	23

### Dialysis

Dialysis has an important role in sodium management. A detailed description of this issue is beyond the scope of the present paper, but is provided elsewhere [30].

To summarize the main points, sodium can be removed in HD and PD by convection and diffusion, and dialysis prescription can be adjusted to optimize sodium removal in both modalities. In HD, ultrafiltration is usually the main mechanism by which sodium removal occurs, but diffusive removal of sodium can be increased by reducing the dialysate sodium concentration [31]. In PD, high volumes and long dwell times are associated with higher sodium removal; a once-daily long-dwell with icodextrin is consistently associated with higher sodium removal and better fluid control in adults [32]. Given that more than 100 mmol of sodium per liter of ultrafiltrate may be lost in PD [33], the relatively higher ultrafiltration rates in infants, the relatively low sodium intake from breastmilk or formula, and the high urinary losses of sodium in some patients may increase the risk for reduced total body sodium chloride content, hypovolemia, hypotension and impaired growth of infants and young children.

### Renal and extra-renal sodium losses

In infants and young children, a chronically uncorrected negative sodium balance is closely related to symptoms of chronic intravascular volume depletion and growth failure [34, 35]. Sodium promotes the expansion of extracellular fluid volume that is necessary for growth, muscle development, and bone mineralization [36]. Infants are especially at risk for the development of a negative sodium balance. This is attributed to their larger relative body surface area compared to their weight, the immaturity of their homeostatic system, and the low sodium content of their feeds (breastmilk contains < 7 mmol/L and infant formula < 15 mmol/L). The increased risk for negative sodium balance is even more prominent in preterm infants, in whom nephron immaturity and the frequent use of diuretics and caffeine cause high urinary sodium losses. Finally, infants and young children with CKD often have kidney diseases associated with polyuria and salt wasting, including kidney dysplasia, obstructive uropathies, and tubulointerstitial nephropathies.

Concomitant extra-renal sodium losses can also predispose infants and children to a negative sodium balance. For instance, children with cystic fibrosis have a sodium and chloride content of sweat that is typically 2–4 times higher than in healthy children [37]. Gastro-intestinal losses of sodium are increased in patients with small bowel ostomies: children with an ileostomy and ileoendorectal pull-through have daily ileal sodium losses between 2 and 3 mmol/kg body weight, often accompanied by signs of intravascular volume depletion [38].

**Q4. What is the sodium requirement for children with kidney diseases?**

## Clinical practice points

4.1 Sodium needs should be determined based on a variety of clinical parameters, aligning with two established reference standards for healthy infants and children:

Adequate Intake (AI): An estimated sodium intake sufficient for healthy individuals when a population reference intake cannot be determined.

Chronic Disease Risk Reduction (CDRR): A sodium intake threshold for individuals > 1 year of age to mitigate the risk of non-communicable diseases, including hypertension, cardiovascular disease, stroke, and coronary heart disease (ungraded).

4.2 For infants under 1 year of age who do not have significant renal or extra-renal sodium losses, target the Adequate Intake for sodium defined for healthy infants as a starting point (grade D, weak).
4.3 For children above 1 year of age, target a sodium intake not exceeding the Chronic Disease Risk Reduction as a starting point (grade D, weak).
4.4 Strict adherence to Adequate Intake (< 1 year of age) and Chronic Disease Risk Reduction (> 1 year of age) is recommended in children with:

- Hypertension or high-normal blood pressure, or those on anti-hypertensive medications (grade A, strong)
- Chronic kidney disease stage 2–4 with blood pressure above 50^th^ percentile (grade B, moderate)
- Albuminuria (grade B, moderate)
- Fluid restriction (grade C, moderate)
- Corticosteroid treatment (grade A, strong)

4.5 Consider increasing sodium intake in the following instances:

- Children with renal or extra-renal sodium losses (grade X, strong)
- Children with hypotension or symptomatic hypovolemia (grade X, strong)
- Infants and toddlers with growth failure resulting from sodium wasting (grade C, weak)
- Children on peritoneal dialysis who have large losses of sodium in the dialysate (grade C, weak)

### Energy and sodium intake

## Key evidence points

4.6 Children who have a high energy requirement are likely to have a higher sodium consumption, even when high sodium foods are limited (grade D, weak).

## Clinical practice points

4.7 In patients with a high energy requirement and/or a poor appetite, higher sodium foods may be permitted on an individual basis, and with careful monitoring of their fluid status and blood pressure (grade D, weak).

## Rationale

The recommendations for sodium intake in children with kidney diseases are based on international guidelines for healthy children, factors influencing sodium sensitivity (see below), and the findings of studies that examined sodium intake in children with kidney diseases, healthy children, and adults with kidney diseases (summarized in Table 3 and Supplementary Table 3).

**Table 3:** Published studies investigating dietary sodium intake in children with kidney diseases (all studies), healthy children (meta-analyses) and adults with kidney disease (meta-analyses)

**STUDIES IN CHILDREN WITH KIDNEY DISEASES**						
**Author, Year**	**Design**	**Patients**	**N**	**Country**	**Methods**	**Outcomes reported**
Parekh, 2001 [34]	Retrospective	Polyuric chronic renal insufficiency (creatinine clearance <65 ml/min/1.73 m^2^)	24	USA	Low-caloric-density, high-volume, sodium-supplemented feedings. Compared with national historic population control from the US Renal Data System database (n=42), and literature controls (n=12).	The change in SDS (Delta SDS) for height by regression analysis at 1 year was significantly greater by +1.37 in the treatment group vs. the population control (p=0.017). The 2-year height Delta SDS by regression analysis adjusted for creatinine clearance was significantly greater by +1.83 in the treatment group vs. the literature control (p=0.003).
Chen, 2017 [4]	Prospective Cross-sectional	CKID Study; age 1–16 years, eGFR 30-90 ml/min/1.73 m^2^	658	USA	Cross-sectional analysis. Food frequency questionnaires	Median sodium intake was 83 (IQR: 55 to 122) mg/kg/day, which is higher than the recommended level in all age groups. Fast foods were the largest single source of sodium, contributing 9.4% of total sodium intake.
Paglialonga, 2023 [29]	Prospective Cross-sectional	HD and PD	41	Europe	Cross-sectional analysis; 3-day dietary diary	Median total sodium intake was 1.5 (IQR 1.2 to 2.3) mEq/kg/day (=60.6% of the maximum recommended intake for healthy children). Median simplified sodium balance (sNaB, defined as intake – urine excretion) was 0.9 (IQR: −0.05 to 1.7) mEq/kg/day. sNaB was the strongest predictor of IDWG in multiple regression analysis. A sNaB ≤0.7 mEq/kg/day and a total sodium intake ≤1.5 mEq/Kg/day were associated with a probability lower than 10% of IDWG ≥4%.
**META-ANALYSIS IN THE GENERAL PEDIATRIC POPULATION**						
**Author, Year**	**Main study question**		**Studies (N of patients)**		**Conclusion**	
He FJ, 2006 [47]	To assess the effect of reducing salt intake on BP in children		10 trials of children and adolescents (966 participants) with a median age of 13 (range: 8–16) years. Salt intake was reduced by 42% (IQR: 7% to 58%) for a median duration of 4 weeks (range: 2 weeks to 3 years).		A modest reduction in salt intake resulted in significant reductions in BP: systolic BP decreased by −1.17 mmHg (95% CI: −1.78 to −0.56) and diastolic BP: −1.29 mmHg (95% CI: −1.94 to −0.65)	
Aburto, 2013 [1]	To assess the effect of decreased sodium intake on BP, related cardiovascular diseases, and potential adverse effects such as changes in blood lipids, catecholamine levels, and renal function in adults and children		9 controlled trials (1299 children and adolescents aged 2.6–19.8 years) and 1 cohort study (596 children aged 5–17 years at baseline and followed for seven years)		9 controlled trials: reducing sodium intake decreased resting systolic BP by 0.84 mmHg (95% CI: 0.25 to 1.4) and diastolic BP by 0.87 mmHg (95% CI: 0.14 to 1.60) in children. The cohort study reported a non-significant higher rate of increase in BP over time in the group of children consuming the highest amount of sodium compared with the group consuming the lowest.	
Leyvraz M, 2018 [2]	Association between sodium intake and BP in healthy children and adolescents		85 studies (14 experimental; 71 observational, including 60 cross-sectional, 6 cohort and 5 case-control studies) with 58,531 participants. 18 experimental and observational studies (3406 participants) of high quality, of which 10 were RCT		In experimental studies, sodium reduction interventions decreased systolic BP by 0.6 mmHg (95% CI: 0.5 to 0.8) and diastolic BP by 1.2 mmHg (95% CI: 0.4 to 1.9). The meta-analysis of 18 experimental and observational studies (with 3406 participants) with sodium intake and BP measurement methods of high quality showed that, for every additional gram of sodium intake per day, systolic BP increased by 0.8 mmHg (95% CI: 0.4 to 1.3) and diastolic BP by 0.7 mmHg (95% CI: 0.0 to 1.4).	
Emmerik, 2020 [48]	To assess the relationship between the amount of sodium intake during the first 6 months after birth and the health effects and/or risk to cardiovascular disease later in life		25 articles including 3 RCTs in humans (11 review studies, 8 RCT’s, 5 prospective cohort studies, and 1 retrospective cohort study; both human and animal studies included)		A relation was found between early salt intake and salt acceptance and/or preference in adulthood. Early dietary sodium intake seems to affect later BP.	
**META-ANALYSIS IN ADULTS WITH KIDNEY DISEASE**						
**Author; Year**	**Main study question**		**N of studies** **(N of patients)**	**Effects on blood pressure**	**Effects on proteinuria**	
D’Elia, 2015 [49]	To investigate the effect of dietary sodium restriction with or without concomitant renin–angiotensin–aldosterone system-inhibiting treatment on albuminuria		11 studies (516 CKD patients) with a follow-up time of 1–6 weeks. Included subjects were healthy (n=1 study), diabetics (n=4), proteinuric (n=2) or hypertensive (n=5). Medications used were ARB and/or ACEi combined (yes/no) with HCT	A greater reduction of urinary albumin excretion was associated with a higher decrease in BP during the intervention.	An average reduction in sodium intake of 92 mmol/d was associated with a reduction in urinary albumin excretion (−32.1% (95% CI: −44.3 to −18.8). The effect of sodium restriction was higher in the cohorts including patients on concomitant renin–angiotensin–aldosterone system-blocking therapy, in the studies with intervention lasting at least 2 weeks, and among participants with evidence of kidney damage.	
Garofalo, 2018 [50]	To evaluate the effects of low vs. high salt intake in adult patients with non-dialysis CKD on change in BP, albuminuria and proteinuria		11 studies (738 CKD patients)	Moderate salt restriction significantly reduces BP in patients with CKD stages 1–4.	Moderate salt restriction significantly reduces proteinuria/albuminuria in patients with CKD stages 1–4.	
McMahon, 2021 (Cochrane review)* [3]	To evaluate the benefits and harms of altering dietary salt for adults with CKD		21 studies (1197 patients): 12 in the earlier stages of CKD (779 patients), 7 in dialysis (363 patients) and 7 in post-transplant (55 patients).	Reducing salt by mean 73.51 mmol/day (95% CI: 92.76-54.27), equivalent to 4.2 g/day or 1690 mg of sodium/day, reduced systolic/diastolic BP by −6.91/−3.91 mmHg (95% CI −8.82 to −4.99/−4.80 to −3.02; 19 studies, 1405 participants; high certainty evidence).	Albuminuria was reduced by 36% (95% CI: 26–44) in 6 studies, five of which were carried out in people in the earlier stages of CKD (mean difference −0.44 mg/day (95% CI: −0.58 to −0.30; 501 participants; high certainty evidence).	
Rozga, 2021 [51]	To examine the effect of sodium-specific medical nutrition therapy provided by renal dietitian on BP, urinary sodium in individuals with CKD 2–5, on dialysis and post-transplant		2000–2020, 8 studies (539 patients): 6 studies in the earlier stages of CKD (437 patients), 2 on maintenance HD (70 patients) and 1 post-transplant (32 patients)	Sodium-specific medical nutrition therapy significantly reduced systolic BP (mean difference: −6.7 mmHg (95% CI: −11.0 to −2.4; I^2^ = 51%) and diastolic BP (−4.8 mmHg (95% CI: −7.1 to −2.4); I^2^ = 23%) as well as urinary sodium excretion (−67.6 mmol/day (95% CI: −91.6 to −43.6); I^2^ = 84.1%). Efficacy was limited to individuals who were not dialyzed, including post-Tx. The intervention did not significantly improve BP in individuals on HD.	--	
Chen, 2022 [52]	To explore the effects of restricting sodium intake on albuminuria and BP in diabetic CKD patients with albuminuria		12 RCTs: 7 for BP (512 diabetic patients)	Salt-restriction diet interventions led to a pooled reduction in systolic BP of −4.72 mmHg (95% CI: −6.71 to −2.73) and in diastolic BP of −2.33 mmHg lower (95% CI: −3.61 to −1.05).	In patients with microalbuminuria, restricted sodium intake decreased albumin excretion rate by 12.62 mg/min (95% CI: −19.64 to −5.60)	
Hodson, 2023 (Cohrane review) [53]	To evaluate the effect of altered salt intake on BP and markers of cardiovascular disease and of CKD in diabetes mellitus patients.		13 RCTs (313 patients with diabetes mellitus types 1 (n=99) and 2 (n=214). Studies were included when the difference in low and high sodium intakes was >34 mmol/day. All but 2 RCTs included patients with normal only.	In both long-term (n=7) and short-term (n=5) studies, sodium intake reduction lowered systolic BP by −6.2 mmHg (95% CI: −9.3 to −3.0) and −8.4 mmHg (95% CI: −14.4 to −2.5) respectively, diastolic BP by −3.4 mmHg (95% CI: −5.56 to −1.27) and –2.95 mmHg (95% CI: −5.0 to −0.94). Mean arterial pressure decreased by 4.60 mmHg (95% CI: −7.26 to −1.94) and 2.37 mmHg (95% CI −4.75 to −0.01) in long-term (n=4) and short-term (n=9) studies, respectively.	Eight studies provided measures of urinary protein excretion before and after salt restriction; four reported a reduction in urinary albumin excretion with salt restriction.	

^*^Previous Cochrane reviews not mentioned in Table: Suckling [2010], McMahon [2015], Palmer [2017]

*ACEi*, angiotensin-converting enzyme inhibitor; *ARB*, angiotensin receptor blocker; *BP*, blood pressure; *CI*, confidence interval; *CKD*, chronic kidney disease; *GFR*, glomerular filtration rate; *HCT*, hydrochlorothiazide; *HD*, hemodialysis; *IDWG*, interdialytic weight gain; *IQR*, interquartile range; *PD*, peritoneal dialysis; *RCT*, randomized controlled trial; *SDS*, standard deviation score; *Tx.*, kidney transplant

### Recommended intake in healthy children

There is no specific biomarker that can be used to establish an average requirement for sodium intake [8, 39–42]. Balance studies for sodium intake in children are limited to a small randomized cross-over study in females 11–15 years of age [43].

Given that a population reference intake, based on an average requirement, cannot be established due to a lack of data, an AI, based on observations from healthy people, has been estimated by various health authorities (Supplementary Table 3). For infants aged 0–6 months, the AI is based on the estimated sodium intake from an average amount of breastmilk [39, 41, 42]. For older infants, 7–12 months, the AI is derived from the combined sodium intake from breastmilk and complementary foods [39, 41], or by extrapolating the 0–6 months AI according to changes in body weight or energy requirements and metabolic body weight [8, 42]. For children older than 1 year, the AI is extrapolated from adult data based on age-specific energy requirements [39, 41, 42] or differences in body weight and a growth factor based on protein requirement to take into account requirements for growth or average energy requirement and a growth factor [8].

The World Health Organization (WHO) and the Nordic countries do not provide an AI but recommend a maximum sodium intake for children aged over 2 years, focusing on the reduction of non-communicable diseases. For children under 16 years, this recommendation is aimed specifically at controlling BP, while for adolescents aged 16–18 years, the focus is on reducing BP and the risk for cardiovascular disease, stroke, and coronary heart disease [39–41]. This sodium intake, referred to as the Chronic Disease Risk Reduction intake (CDRR), is shown in Supplementary Table 4. The European Food Safety Authority (EFSA) has adopted a similar approach for children over 1 year, referring to it as the Safe Intake (SI), which reflects the relationship between sodium intake and the risk for cardiovascular disease [8]. The CDRR for children is extrapolated from the adult CDRR using differences in energy requirements. In contrast, New Zealand and Australia have established an Upper Limit (UL), representing the highest average intake that does not pose a risk to the general population [42].

### Factors affecting sodium sensitivity

Sodium sensitivity refers to an increase in BP in response to sodium loading [44]. The kidney has a key role in the development of sodium sensitivity through pressure natriuresis [44]. In individuals with sodium sensitivity, the natriuretic response is inadequate, leading to sodium retention and BP elevation: CKD is therefore widely accepted as a major risk factor for sodium-sensitive hypertension. Sodium intake leads to higher blood pressure increases in patients with reduced nephron mass, such as children with a congenital solitary kidney, compared to those with normal nephron mass [45]. In recent years, novel mechanisms of this blood pressure phenotype have been identified, that involve the non-osmotic sodium stores, the gut microbiome, the immune system, and epigenetic modifications.

Some other factors, including prematurity, obesity, and African ancestry, may confer sodium sensitivity [44, 46]. In a cross-sectional study using ambulatory BP monitoring, sodium sensitivity was present in 37% and 47% of the low birth weight and small for gestational age children at early adolescence and correlated with kidney length [47].

In a weight loss intervention study, salt restriction led to an average mean BP reduction of 12 mmHg in obese children compared to a 1 mmHg rise in the normal weight group, while weight loss lessened sodium sensitivity [48]. Further observational studies supported the role of sodium sensitivity in overweight and obese children [49]. The most robust evidence comes from a National Health and Nutrition Examination Survey study of 6235 children and adolescents: mean adjusted systolic BP increased progressively with sodium intake quartile; this association was stronger among those who were overweight/obese [49].

### Recommended intake for children with kidney diseases

Children with kidney diseases are a highly heterogeneous population in terms of sodium needs, making a universal approach inappropriate. In many children with kidney diseases, a recommended intake identical to that of healthy peers is reasonable in the absence of any clinical or laboratory findings to suggest different requirements (Table 4).

**Table 4:** Recommended sodium intake in children with kidney disease per age group

**Age < 1 year**	Adequate intake* of sodium (g/day)	Adequate intake* of salt (NaCl) (g/day)
0–3 m	0.11–0.12	0.28–0.30
4–6 m	0.11–0.20	0.28–0.50
7–11 m	0.17–0.37	0.43–0.90
**Age > 1 year**	CDRR for sodium intake** **(g/day)**	CDRR for salt intake** **(g/day)**^**#**^
1–3 y	1.2	3.0
4–6 y	1.5	3.5
7–8 y	1.7	4.0
9–13 y	2.0	5.0
14–18 y	2.3	5.5

Definitions

^*^Adequate Intake (AI): an estimated sodium intake sufficient for healthy individuals when a population reference intake cannot be determined

^**^based on Chronic Disease Risk Reduction (CDRR): a sodium intake threshold for individuals to mitigate the risk of noncommunicable diseases, including hypertension, cardiovascular disease, stroke, and coronary heart disease

m, month; y, year

^#^for practical use rounded down to the nearest whole or half gram per day

While AI could be considered a feasible and appropriate target for sodium intake in infants, it is difficult to achieve a well-balanced diet for children over 1 year of age if targeting the AI. A sodium intake below the CDRR (as for healthy peers) is a reasonable and practical target to reduce the risk of hypertension and cardiovascular morbidity for children over 1 year of age. The CDRR for salt intake is significantly lower than the actual salt intake of children with CKD [4].

Reducing sodium intake below the CDRR is an important preventive measure for cardiovascular disease in the general population; however, it is especially important in patients with kidney diseases, particularly those with or at risk for hypertension and fluid overload. The role of a low-salt diet in reducing BP has been demonstrated in many RCTs and meta-analyses both in healthy individuals and adults with kidney diseases (Table 2 and Supplementary Table 5), with a greater effect in CKD patients than in the general population [1–3, 50–56]. Sodium intake reduction should therefore be recommended as a first-line treatment option in children with hypertension, high-normal BP, or receiving anti-hypertensive medications. The same is true for children with CKD 2–4 and BP above the 50th percentile, given that a reduction of BP below this threshold can slow the progression of CKD [57].

Some RCTs in adults have shown that reducing sodium intake is associated with a reduction in albuminuria, a known risk factor for CKD progression [3, 52, 53].

Renin–angiotensin–aldosterone system inhibitors (RAASi), commonly used both to reduce BP and proteinuria, seem to have a synergistic effect with dietary sodium reduction. Post-hoc analysis of the “Reduction of Endpoints in Non-insulin dependent diabetes mellitus with the Angiotensin II Antagonist Losartan” (RENAAL) and “Irbesartan Diabetic Nephropathy” (IDNT) showed that the cardiovascular and kidney benefits of angiotensin receptor blockade were only realized in patients with the lowest quartile of sodium intake [58]. A meta-analysis of trials in adults showed that sodium restriction reduced albuminuria with a greater effect size in patients taking RAASi [52].

Sodium affects BP through several mechanisms, but its impact on fluid status remains the most significant. Thirst is mainly an osmotic-driven process, which explains why sodium intake is the main determinant of fluid intake. A recent observational trial demonstrated that interdialytic sodium balance is the strongest independent predictor of IDWG in children on HD [29]. A reduction in sodium intake should always be recommended when a reduction of fluid intake is prescribed.

Corticosteroids increase tubular sodium reabsorption, thereby increasing the risk of high BP. Reducing sodium intake should be suggested to treat and prevent sodium and fluid overload in patients receiving these drugs, despite the lack of trials investigating this approach.

Although primary tubulopathies are excluded from these CPRs according to the PICO questions, the role of sodium restriction in some patients with polyuria to reduce urine output needs also to be mentioned.

In the absence of sound scientific evidence, the target sodium intake in children with high BP, fluid overload, albuminuria, or receiving corticosteroids is under debate. Given that sodium intakes well below the CDRR cannot be achieved without compromising the quality of the diet, it is the opinion of the PRNT that targeting the AI for infants and keeping sodium intake below the CDRR for older children is safe and effective even in children with kidney disease at the highest risk of cardiovascular consequences. In these cases, the benefit of sodium restriction consists not only in the prevention of long-term cardiovascular sequelae, but also in a significant immediate improvement in clinical parameters, such as BP and fluid status. The recommended intakes and the indications for sodium restriction are fully consistent with the 2020 National Kidney Foundation’s Kidney Disease Outcomes Quality Initiative (KDOQI) clinical practice recommendations for adults with CKD [59].

### Children with kidney diseases needing increased sodium intake

In children with hypotension, symptomatic hypovolemia, or growth failure associated with sodium wasting, an increase in sodium intake is needed. Children at highest risk are infants and toddlers with polyuric salt-wasting kidney diseases, patients who receive PD, or children with extra-renal sodium losses (e.g., small bowel ostomies, cystic fibrosis).

The literature on this topic is limited. Parekh et al. 2001 demonstrated in 24 infants (age < 1 year) with salt-wasting CKD that high-volume and sodium-supplemented feedings can significantly improve growth [35].

Given wide variations in sodium losses across different salt-wasting diseases and from PD, it is not possible to suggest a specific intake that would apply to every patient. Instead, the dietary prescription and the supplementation must be individualized, with attention directed particularly at BP and growth.

### Energy and sodium intake

Children with a higher energy intake have usually a higher sodium intake, even when prescribed low-sodium foods. Given the importance of preventing malnutrition and optimizing growth, a sodium intake above the CDRR may be accepted in some patients with low appetite and/or high energy needs to obtain an adequate calorie and protein intake; this should be decided on an individual basis, with careful monitoring of fluid status and BP.

**Q5. How to reduce sodium intake in children with kidney diseases?**

## Clinical practice points

5.1 Educate the child and family regarding a lower-sodium diet early in the course of kidney disease (grade C, moderate).
5.2 Educate the family to reduce sodium intake from early childhood to avoid developing a taste preference for higher sodium foods (grade C, moderate).
5.3 Consider reducing sodium intake from non-dietary sources, including medications and the dialysis prescription, to avoid nutritional compromise (grade C, moderate).
5.4 Reduce consumption of processed and ultra-processed food and encourage low-sodium unprocessed foods (grade C, moderate).
5.5 Select lower-sodium alternatives when foods are processed or ultra-processed (grade C, moderate).
5.6 Avoid salt substitutes in children with impaired kidney function (grade B, moderate).

## Rationale

Reducing sodium intake in children with kidney diseases is challenging, especially for those with reduced kidney function, as impaired salt taste sensitivity in patients with CKD makes it difficult for them to reduce their salt intake [60]. Early intervention and education on dietary sodium reduction are essential for children with kidney diseases. Healthcare providers, particularly pediatric nephrologists and dietitians, play a vital role in early education pertaining to sodium intake. By understanding the risks associated with high sodium intake and learning some practical strategies to reduce sodium consumption, caregivers can avoid or reduce the consumption of high-sodium foods in the child’s diet, ideally before they become habituated. Education should begin as soon as a child is diagnosed with kidney disease and focus on the major sources of sodium, such as processed foods, fast foods, cured meat, and snacks. This can help families make changes to the child’s diet, and ideally to the diet of the entire family. An early education to a healthy diet without sodium excess is important even in patients with sodium wasting, as some of them may experience high BP with typical westernized diets despite increased sodium losses [4]. This can also make it easier for them to adapt to any changes in their sodium needs that may occur over the course of their kidney disease.

A key strategy for reducing sodium intake is the promotion of cooking meals at home and limiting the number of processed and ultra-processed foods. Encouraging families to cook with fresh ingredients instead of relying on processed foods can dramatically lower sodium intake [61]. This may involve a self-care skills program to teach caregivers how to cook, and support life skills for children and young people [62, 63].

Dietary habits formed early in life are likely to track later in childhood and form the basis for adult eating patterns. Early nutrition education should focus on incorporating easy to prepare, lower sodium, kidney-friendly recipes that use herbs, spices, and other low-sodium flavoring options, which provide a healthy alternative to salt.

Package labeling for sodium or salt includes logos and symbols (see Supplementary Fig. 2), nutrient claims (Table 1), and sodium content listings, which may vary by country. Educating children and their caregivers on how to read and interpret nutritional labels enables the selection of lower-sodium options and can achieve about a 35% reduction in salt intake [64].

**Fig. 3:**
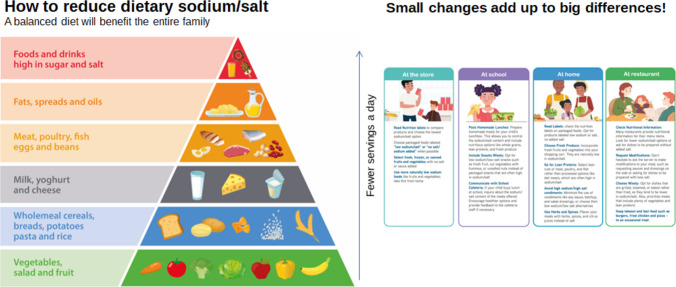
Infographic on how to reduce dietary sodium/salt

Figure 3 gives a pyramid representation of a balanced diet, which can be used by healthcare professionals to educate the whole family regarding a healthy lower-sodium diet. Ideally, this education should begin early on in the course of the child’s kidney disease.

The use of low-sodium salt substitutes (LSSS) containing potassium chloride is a potential strategy for reducing sodium intake; however, there is currently no evidence supporting the effectiveness of LSSS in lowering BP in children. Most importantly, due to the lack of evidence on the safety of LSSS use in children and the potential risk of hyperkalemia, particularly in children with kidney diseases [65], the use of LSSS is not recommended for patients with CKD.

There is evidence that salt taste thresholds decrease with reduced sodium intake, with high-salt foods becoming unpalatable within 4–6 weeks of following a low-sodium diet [66, 67]. It is important to communicate this adaptation period to families.

Sodium restriction poses a potential concern for reduced iodine intake, as iodized salt is a primary source of dietary iodine in certain countries. Iodine deficiency can lead to hypothyroidism, impaired growth, and neurological and cognitive deficits [68]. Countries that rely on iodine fortification strategies, such as iodized table salt and iodized salt in food production and bread-making (e.g., the Netherlands, Belgium, and Denmark), may have an increased risk of iodine deficiency under sodium reduction measures. No trials investigated iodine status in children with kidney diseases. Since iodine accumulates with reduced kidney function, iodine deficiency in children with CKD may not be a risk even with a low consumption of iodized salt [69].

**Q6. How to achieve adequate sodium intake in children with increased sodium requirements?**

## Clinical practice points

6.1 Adapt the prescription in children on dialysis to optimize the sodium balance (grade D, weak).
6.2 Increase dietary sodium intake, maintaining a varied and balanced diet (grade D, weak).
6.3 Introduce and titrate sodium supplements to achieve euvolemia and optimize growth (grade D, weak).

## Rationale

All the possible factors impacting on sodium balance should be taken into account when increasing sodium intake, including the dialysis prescription. A sodium intake higher than the CDRR may be needed, but a variety of food sources and a high-quality diet should always be guaranteed.

The addition of sodium to the diet, feeds, or formula may be required. The best option in this case is to administer a commercially available sodium chloride solution. Sodium chloride solution can also be prepared inexpensively at home by dissolving a specific quantity of table salt in a known volume of water, but some safety concerns need to be considered. The concentration and dosage volumes can be adjusted according to the specific clinical requirements. Taking into account that each gram of sodium chloride contains 400 mg of sodium (= 17 mmol), it is possible, for example, to prepare a 1 mmol/ml sodium solution by adding 5.8 g of sodium chloride (1 teaspoon of table salt) to 100 ml of water (Supplementary Table 6). The prepared sodium solution may be administered orally, in combination with a varied and balanced diet, or as an addition to feeds taken orally or through enteral feeding tubes. The volume and frequency of administration will vary depending on the clinical goals, the patient’s sodium requirements, and individual tolerance. Careful preparation, administration, and monitoring are essential to ensure that sodium supplementation is effective and safe for achieving euvolemia and optimal growth; possible differences in the concentration of sodium of the salt formulations used should be considered. The possible risks associated with an improper preparation of the solution should be anticipated.

## Summary of recommendations

Table 5 shows a summary of recommendations.

**Table 5:** Summary of statements

**Q1. What are the main dietary sources of sodium for an infant, child and adolescent?**		
Key evidence points		
1.1	Sodium intake varies across regions and between countries due to cultural differences in consumption, cooking practices, taste preferences and differences in food-processing methods	Ungraded
1.2	The major sources of sodium are processed and ultra-processed foods through the use of added salt and sodium-containing additives	Grade B, moderate
**Q2. How is dietary sodium intake assessed in children?**		
Clinical practice points		
2.1	We suggest using a diet recall of a typical 24-h period or a food frequency questionnaire to identify the main dietary sources of sodium	Grade C, weak
2.2	We suggest using a 3-day prospective diet diary for an in-depth analysis of sodium intake	Grade C, weak
2.3	We do not recommend the routine measurement of serum sodium or urinary sodium to estimate the sodium intake or sodium balance in children with kidney diseases	Grade C, weak
**Q3. What are the non-dietary factors contributing to sodium balance?**		
Key evidence points		
3.1	Medications can be a significant source of sodium	Ungraded
3.2	Dialysis can lead to a gain or loss of sodium depending on the individual’s sodium balance and the dialysis prescription	Grade C, moderate
3.3	Salt-wasting kidney diseases and extra-renal losses may lead to sodium depletion in infants and young children	Grade C, moderate
**Q4. What is the sodium requirement for children with kidney diseases?**		
Clinical practice points		
4.1	Sodium needs should be determined based on a variety of clinical parameters, aligning with two established reference standards for healthy infants and children: -Adequate Intake (AI): An estimated sodium intake sufficient for healthy individuals when a population reference intake cannot be determined -Chronic Disease Risk Reduction (CDRR): A sodium intake threshold for individuals > 1 year of age to mitigate the risk of noncommunicable diseases, including hypertension, cardiovascular disease, stroke, and coronary heart disease	Ungraded
4.2	For infants under 1 year of age who do not have significant renal or extra-renal sodium losses, target the Adequate Intake for sodium defined for healthy infants as a starting point	Grade D, weak
4.3	For children above 1 year of age, target a sodium intake not exceeding the Chronic Disease Risk Reduction as a starting point	Grade D, weak
4.4	Strict adherence to Adequate Intake (< 1 year of age) and Chronic Disease Risk Reduction (> 1 year of age) is recommended in children with	
	- hypertension or high-normal blood pressure, or those on anti-hypertensive medications	Grade A, strong
	- chronic kidney disease stage 2–4 with blood pressure above 50th centile	Grade B, moderate
	- albuminuria	Grade B, moderate
	- fluid restriction	Grade C, moderate
	- corticosteroid treatment	Grade A, strong
4.5	Consider increasing sodium intake in the following instances	
	- children with renal or extrarenal sodium losses	Grade X, strong
	- children with hypotension or symptomatic hypovolemia	Grade X, strong
	- infants and toddlers with growth failure resulting from sodium wasting	Grade C, weak
	- children on peritoneal dialysis who have large losses of sodium in the dialysate	Grade C, weak
**Energy and sodium intake**		
Key evidence points		
4.6	Children who have a high energy requirement are likely to have a higher sodium consumption, even when high-sodium foods are limited	Grade D, weak
Clinical practice points		
4.7	In patients with a high energy requirement and/or a poor appetite, higher sodium foods may be permitted on an individual basis, and with careful monitoring of their fluid status and blood pressure	Grade D, weak
**Q5. How to reduce sodium intake in children with kidney diseases?**		
Clinical practice points		
5.1	Educate the child and family regarding a lower-sodium diet early in the course of kidney disease	Grade C, moderate
5.2	Educate the family to reduce sodium intake from early childhood to avoid developing a taste preference for higher sodium foods	Grade C, moderate
5.3	Consider reducing sodium intake from non-dietary sources, including medications and the dialysis prescription, to avoid nutritional compromise	Grade C, moderate
5.4	Reduce consumption of processed and ultra-processed food and encourage low-sodium unprocessed foods	Grade C, moderate
5.5	Select lower-sodium alternatives when foods are processed or ultra-processed	Grade C, moderate
5.6	Avoid salt substitutes in children with impaired kidney function	Grade B, moderate
**Q6. How to achieve adequate sodium intake in children with increased sodium requirements?**		
Clinical practice points		
6.1	Adapt the prescription in children on dialysis to optimize the sodium balance	Grade D, weak
6.2	Increase dietary sodium intake, maintaining a varied and balanced diet	Grade D, weak
6.3	Introduce and titrate sodium supplements to achieve euvolemia and optimize growth	Grade D, weak

## Results of the Delphi survey

Forty-five responses were received via an electronic Delphi survey, including 26 dietitians and 32 pediatric nephrologists from 24 countries. Delphi respondents are listed as “Delphi survey participants” in the Acknowledgements section. The 24 statements received an overall average consensus of 89.9% with a “strongly agree” or “agree” response and 8.4% with a “neutral” response. The response was “disagree” in 1.7% of cases, while no statements received a “strongly disagree” response. All but one of the statements met the required level of agreement of 70% or more. One statement received a 69% of agreement, with 22% “neutral” and 9% disagreeing (statement 2.3). Specifically, a small number of reviewers argued that urinary sodium measurement may be helpful in some circumstances; the statement was partially revised according to their comments, and the rationale was expanded to better clarify the available evidence.

Despite receiving 91% agreement, a minor change was made to statement 4.1 following reviewers’ comments to better emphasize that we suggest the same sodium intake as healthy peers as a reasonable starting point for many children with kidney diseases.

## Research recommendations

- Validation of methods to assess sodium intake in children with kidney diseases.
- Explore the reasons for nonadherence to a sodium-restricted diet in order to develop evidence-based interventions to promote dietary adherence.
- Investigate the effect of sodium restriction on CKD progression.
- Identify the minimum sodium needs in children.
- Identify the optimal dialysate sodium in children on dialysis and the best strategies to optimize sodium removal in PD.
- Investigate the efficacy and safety of LSSS in the early stages of CKD.

## Supplementary Information

Below is the link to the electronic supplementary material.

MOESM 1 (DOCX 911 KB)

## Data Availability

Data sharing is not applicable to this article as no new data were created or analyzed in this study.
