# Supplementary material for: The dietary management of sodium in children with kidney diseases—clinical practice recommendations from the Pediatric Renal Nutrition Taskforce
Source: Pediatr Nephrol. 2025 Nov 18;41(5):1535–56. doi: 10.1007/s00467-025-06913-z (PMC13009053; doi:10.1007/s00467-025-06913-z)
Supplement: Supplementary file 1 — (DOCX 911 KB) [file 467_2025_6913_MOESM1_ESM.docx]

**Supplemental Table 1. Search terms strategy used in the literature review**

| 1 | kidney disease | renal failure | renal insufficiency | chronic kidney disease | kidney failure | kidney injury | kidney dysfunction | CKD |
| --- | --- | --- | --- | --- | --- | --- | --- | --- |
|  | chronic renal failure | chronic kidney failure | ESKD  (and ESRD) | ESRF | dialysis | renal replacement therapy | pre dialysis | peritoneal dialysis |
|  | hemodialysis | hemodialysis | CAPD | APD |  |  |  |  |
| 2 | sodium | salt |  |  |  |  |  |  |
| 3 | diet | dietary | nutrition | food | feed | status | intake | requirements |
|  | dietary management | dietary advice | dietary restriction | Supplemen-tation | dietitian | dietician | constipation | gastrointestinal |

ESKD: End-stage kidney disease

ESRD: End-stage renal disease

ESRF: End-stage renal failure

CAPD: chronic ambulatory peritoneal dialysis

APD: automated peritoneal dialysis

**Supplementary Figure 1. American Academy of Pediatrics grading matrix**


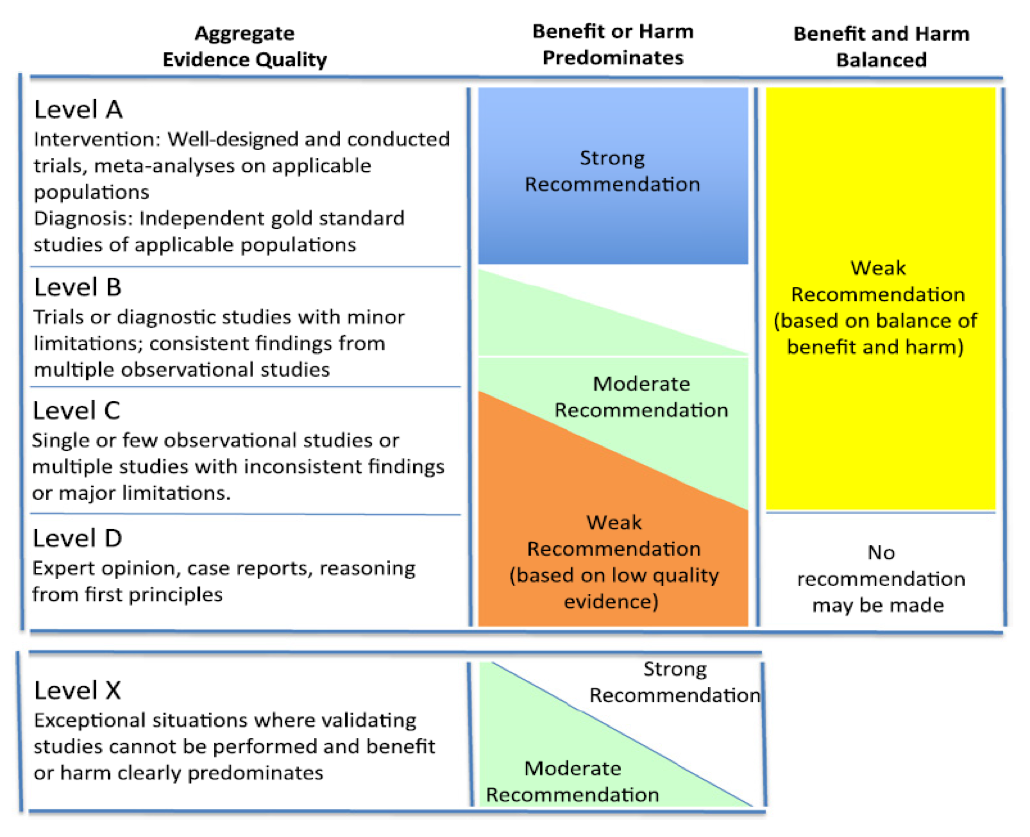


Reference: American Academy of Pediatrics Classifying recommendations for clinical practice guidelines. Pediatrics. 2004;114:874–877. doi: 10.1542/peds.114.2.506.

**Supplementary Table 2. Sodium containing additives**

| **Products *** | **Name** | **E number** |
| --- | --- | --- |
| **Preservative** | | |
| Bread, soft drinks, vegetables or fruit in a can or glass, cookies and pastries, snacks, meat, dietary supplements, dairy products | Sodium acetates | E 262 |
| Alcohol-free beer, soft drinks, boiled beetroot, cooked shellfish, jam, olives, sauces, fruit juice | Sodium benzoate | E 211 |
| Bread, soft drinks, vegetables or fruit in a can or glass, cookies and pastries, snacks, meat, dietary supplements, dairy products | Sodium diacetate | E 262 (ii) |
| Nuts, pate, snacks, sweets, meats | Sodium ethyl p-hydrocybenzoate | E 215 |
| Nuts, pate, snacks, sweets, meats | Sodium methyl p-hydroxybenzoate | E 219 |
| Pickled herring and sprat, cheese | Sodium nitrate | E 251 |
| Meat | Sodium nitrite | E 250 |
| Bread, cheese, cookies and pastries | Sodium propionate | E 281 |
| Caviar | Sodium tetraborate (borax) | E 285 |
| **Food acid** |  |  |
| Desserts, powders for beverages, pie filling | Sodium adipate | E 356 |
| Bread, soft drinks, jam, cookies and pastries, snacks, dietary supplements, sweets, dairy products | Sodium citrates | E 331 |
| Bread, soft drinks, vegetables or fruit in a can or glass, cookies and pastries, snacks, meat, dietary supplements, dairy products | Sodium lactate | E 325 |
| Bread, soft drinks, jam, cookies and pastries, snacks, dietary supplements, dairy products | Sodium malates | E 350 |
| Flower, ice, cookies and pastries, mineral water, oils and fats, meat, salty, dairy products | Sodium phosphate | E 339 |
| Bread, soft drinks, vegetables or fruit in a can or glass, cookies and pastries, snacks, dietary supplements, dairy products | Sodium potassium tartrate | E 337 |
| Bread, soft drinks, vegetables or fruit in a can or glass, cookies and pastries, snacks, dietary supplements, dairy products | Sodium tartrates | E 335 |
| **Thickener** | | |
| Bread, soft drinks, cookies and pastries, pudding, snacks, sweets, dietary supplements, dairy products | Sodium alginate | E 401 |
| Bread, soft drinks, cookies and pastries, snacks, traditional Swedish and Finnish citrus fruit syrup, dietary supplements, sweets, dairy products | Sodium carboxy methyl cellulose, cellulose gum | E 466 |
| Infant food, bread, soft drinks, cookies and pastries, snacks, toddlers food, dietary supplements, dairy products | Starch sodium octenyl succinate | E 1450 |
| **Antioxidant** | | |
| Beer, bread, soft drinks, cookies and pastries, snacks, meat, dietary supplements, dairy products | Sodium ascorbate | E 301 |
| Fish, meat | Sodium erythorbate | E 316 |
| **Acidity-regulator** | | |
| Bread, chocolate, soft drinks, cookies and pastries, snacks, dietary supplements, salt, dairy products | Sodium carbonates | E 500 |
| Bread, chocolate, soft drinks, jam, cookies and pastries, snacks, dietary supplements, dairy products | Sodium hydroxide | E 524 |
| **Complexing agent** | | |
| Canned and jarred vegetables, sauces, shellfish, fats | Calcium disodium enthylene diamene tetra acetate | E 385 |
| Bread, soft drinks, cookies and pastries, snacks, dietary supplements, sweets, dairy products | Sodium gluconate | E 576 |
| **Preservative and antioxidant** | | |
| Alcoholic beverages, grapes, soft drinks, vegetables, cinnamon, cookies, mustard, sugar | Sodium hydrogen sulphite | E 222 |
| Alcoholic beverages, grapes, soft drinks, vegetables, cinnamon, cookies, mustard, sugar | Sodium metabisulphite | E 223 |
| **Flavor enhencer** | | |
| Bread, soft drinks, cookies and pastries, herbs, snacks, dietary supplements, salt substitutes, dairy products | Disodium guanylate | E 627 |
| Bread, soft drinks, cookies and pastries, herbs, snacks, dietary supplements, salt substitutes, dairy products | Disodium inosinate | E 631 |
| Bread, soft drinks, cookies and pastries, herbs, snacks, dietary supplements, salt substitutes, dairy products | Disodium 5-ribonucleotides | E 635 |
| Bread, soft drinks, cookies and pastries, snacks, dietary supplements, sweeteners, dairy products | Glycine and sodium glycinate | E 640 |
| Bread, soft drinks, cookies and pastries, herbs, snacks, dietary supplements, salt substitutes, dairy products | Mono sodium glutamate | E 621 |
| **Others: acidity regulator, antioxidant, (anti)raising agent, carrier, complexing agent, emulsifier, filler, preservative, stabilizer, strengthening agent, thickener** |  |  |
| Candied cherries, liquid egg white for egg foam | Aluminium sodium sulphate | E 521 |
| Cake | Sodium aluminium phosphate acidic | E 541 |
| Salt for the outside of cheese | Sodium aluminium silicate | E 554 |
| Salt, salt substitutes | Sodium ferrocyanide | E 535 |
| Bread, chocolate, soft drinks, cookies and pastries, snacks, dietary supplements, salt, dairy products | Sodium hydrogenate carbonate | E 500 (ii) |
| Bread, soft drinks, dried herbs, cookies and pastries, snacks, dietary supplements, sweets, dairy products | Sodium, potassium and calcium salts of fatty acids | E 470a |
|  |  |  |
| Alcoholic beverages, chewing gum, cookies and pastries, oils and fats, breakfast cereals, quick-cooked rice, meat in a can or glass, dairy products | Sodium stearoyl-2-lactylates | E 481 |
| Bread, soft drinks, cookies and pastries, snacks, dietary supplements, dairy products | Sodium sulphates | E 514 |
| Alcoholic beverages, grapes, soft drinks, vegetables, cinnamon, cookies, mustard, sugar | Sodium sulphite | E 221 |
| Dietary supplements, sweeteners | Vernette sodium carboxy methyl cellulose, vernette cellulose gum | E 468 |

References:

<https://ec.europa.eu/food/food-feed-portal/screen/food-additives/search> Assessed March 2025

*website The Netherlands Nutrition Centre; Assessed March 2025.

**Supplementary Table 3: International recommendations for sodium intake in healthy children: adequate intake**

| **International recommendations for sodium intake in healthy children: Adequate Intake (gram/day)** | | | | | |
| --- | --- | --- | --- | --- | --- |
|  | **NNR 2023** | **EFSA 2019** | **NASEM**  **2019** | **DACH 2016** | **NHMRC**  **2017** |
| **AGE** | **AI** | **AI** | **AI** | **AI** | **AI** |
| 0-3 m | 0.11 g | - | 0.11 g | - | 0.12 g |
| 4-6 m | 0.11 g | - | 0.11 g | 0.20 g | 0.12 g |
| 7-11 m | 0.37 g | 0.20 g | 0.37 g | 0.20 g | 0.17 g |
| 1-3 y | - | - | 0.80 g | 0.40 g | 0.20-0.40 g |
| 4-6 y | - | - | 1.00 g | 0.50 g | 0.30-0.60 g |
| 7-8 y | - | - | 1.00 g | 0.75 g | 0.30-0.60 g |
| 9 y | - | - | 1.20 g | 0.75 g | 0.40-0.80 g |
| 10 y | - | - | 1.20 g | 1.10 g | 0.40-0.80 g |
| 11-12 y | - | - | 1.20 g | 1.10 g | 0.40-0.80 g |
| 13 y | - | - | 1.20 g | 1.40 g | 0.40-0.80 g |
| 14 y | - | - | 1.50 g | 1.40 g | 0.46-0.92 g |
| 15-18 y | - | - | 1.50 g | 1.50 g | 0.46-0.92 g |

AI = Adequate Intake; m = months; y = years

NNR = Nordic Nutrition Recommendations – 2023 (Denmark, Finland, Iceland, Norway and Sweden)^1^

EFSA = European Food and Safety Authority – 2019 (Europe) ^2^

NASEM = National Academies of Sciences, Engineering, and Medicine – 2019 (USA) ^3^

DACH = Deutsche Gesellschaft für Ernährung, Österreichische Gesellschaft für Ernährung, Schweizerische Gesellschaft für Ernährung – 2016 (German/Austria/Switzerland) ^4^

NHMRC = National Health and Medical Research Council – 2006 (Australia/New Zealand); updated 2017 (Australia/New Zealand) ^5^

References

^1^ Blomhoff R., Andersen R, Arnesen E.K., Christensen JJ, Eneroth H, Erkkola M, Gudanavicienne L, Halldorsson TI, Hoyer-Lund A, Lemming EW, Meltzer HM, Pitsi T, Schwab U, Siksna I, Thorsdottier I, Trolle E (2023) Nordic Nutrition Recommendations 2023, Copenhagen: Nordic Council of Ministers.

^2^ EFSA Panel on Nutrition, Novel Foods and Food Allergens (NDA); Turck D, Castenmiller J, de Henauw S, Hirsch-Ernst KI, Kearney J, Knutsen HK, Maciuk A, Mangelsdorf I, McArdle HJ, Pelaez C, Pentieva K, Siani A, Thies F, Tsabouri S, Vinceti M, Aggett P, Fairweather-Tait S, Martin A, Przyrembel H, Ciccolallo L, de Sesmaisons-Lecarré A, Valtueña Martinez S, Martino L, Naska A. (2019) Dietary reference values for sodium. EFSA J 17:e05778

^3^ National Academies of Sciences, Engineering, and Medicine. 2019. Dietary Reference Intakes for Sodium and Potassium. Washington, DC: The National Academies Press. [https://doi.org/10.17226/25353](https://protect.checkpoint.com/v2/r06/___https://doi.org/10.17226/25353___.ZXV3Mjp1bml2ZXJzaXR5aG9zcGl0YWxzb3V0aGFtcHRvbjpjOm86MjhmNjE0OGY0ZTE0MTQ1MTk4OTQyZmMyNDE3NWI4MWI6Nzo2OWFiOmM5YWU5NGY5ZTE2N2M4MTRkNTVjYzc5MDIzNjk0MzdhNjQ5NzJiNjZmNDFiMWVkMGQxYTdiY2NkMDVlNzhiZjM6cDpGOk4)

^4^ Deutsche Gesellschaft für Ernährung, Österreichische Gesellschaft für Ernährung, Schweizerische Gesellschaft für Ernährung, 2015. Referenzwerte für die Nährstoffzufuhr. 2. Auflage, 1. Ausgabe. DGE, Bonn, Germany.

^5^ National Health and Medical Research Council, Australian Government Department of Health and Ageing, New Zealand Ministry of Health. Nutrient Reference Values for Australia and New Zealand. Canberra: National Health and Medical Research Council; 2006, sodium updated 2017

**Supplementary Table 4: International recommendations for sodium intake (expressed as g/day) in healthy children: Chronic Disease Risk Reduction (CDRR) or upper limit (UL) or safe intake (SI)**

| **International recommendations for sodium intake in healthy children: Chronic Disease Risk Reduction, Upper Limit or Safe Intake (gram/day)** | | | | | |
| --- | --- | --- | --- | --- | --- |
| **AGE** | **NNR**  **2023** | **WHO**  **2012** | **EFSA**  **2019** | **NASEM**  **2019** | **NHMRC**  **2017** |
|  | **CDRR** | **CDRR** | **SI** | **CDRR** | **UL** |
| 0-3 m | - | - | - | - | - |
| 4-6 m | - | - | - | - | - |
| 7-11 m | - | - | - | - | - |
| 1-2 y | 1.10 g | - | 1.10 g | 1.20 g | 1.00 g |
| 2-3 y | 1.10 g | 2.00 g/day adjusted downward based on energy requirements of children relative to those of adults (*) | 1.10 g | 1.20 g | 1.00 g |
| 4-6 y | 1.40 g |  | 1.30 g | 1.50 g | 1.40 g |
| 7-8 y | 1.70 g |  | 1.70 g | 1.50 g | 1.40 g |
| 9 y | 1.70 g |  | 1.70 g | 1.80 g | 2.00 g |
| 10 y | 1.70 g |  | 1.70 g | 1.80 g | 2.00 g |
| 11-12 y | 2.00 g |  | 2.00 g | 1.80 g | 2.00 g |
| 13 y | 2.00 g |  | 2.00 g | 1.80 g | 2.00 g |
| 14 y | 2.00 g |  | 2.00 g | 2.30 g | 2.30 g |
| 15-18 y | 2.30 g |  | 2.00 g | 2.30 g | 2.30 g |

CDRR = Chronic Disease Risk Reduction; UL = Upper Limit; SI = Safe Intake; m = months; y = years

NNR = Nordic Nutrition Recommendations – 2023 (Denmark, Finland, Iceland, Norway and Sweden) ^1^

WHO = World Health Organization – 20126 ^2^

EFSA = European Food and Safety Authority – 2019 ^3^

NASEM = National Academies of Sciences, Engineering, and Medicine - 2019 (USA) ^4^

NHMRC = National Health and Medical Research Council – 2006 (Australia/New Zealand); updated 2017 (Australia/New Zealand) ^5^

(*) Adjusting the sodium intake downward based on the requirement of children relative to those of adults: each country should determine the energy requirements of various age categories of the paediatric population relative to adults approximately 20-50 years of age, to lower the recommended maximum intake value of 2 g/day.

References

^1^ Blomhoff R., Andersen R, Arnesen E.K., Christensen JJ, Eneroth H, Erkkola M, Gudanavicienne L, Halldorsson TI, Hoyer-Lund A, Lemming EW, Meltzer HM, Pitsi T, Schwab U, Siksna I, Thorsdottier I, Trolle E (2023) Nordic Nutrition Recommendations 2023, Copenhagen: Nordic Council of Ministers

^2^World Health Organization Nutrition. Guideline: Sodium Intake for Adults and Children; World Health Organization (WHO): Geneva, Switzerland, 2012; ISBN 9789241504836.

^3^ EFSA Panel on Nutrition, Novel Foods and Food Allergens (NDA); Turck D, Castenmiller J, de Henauw S, Hirsch-Ernst KI, Kearney J, Knutsen HK, Maciuk A, Mangelsdorf I, McArdle HJ, Pelaez C, Pentieva K, Siani A, Thies F, Tsabouri S, Vinceti M, Aggett P, Fairweather-Tait S, Martin A, Przyrembel H, Ciccolallo L, de Sesmaisons-Lecarré A, Valtueña Martinez S, Martino L, Naska A. (2019) Dietary reference values for sodium. EFSA J 17:e05778

^4^ National Academies of Sciences, Engineering, and Medicine. 2019. Dietary Reference Intakes for Sodium and Potassium. Washington, DC: The National Academies Press. [https://doi.org/10.17226/25353](https://protect.checkpoint.com/v2/r06/___https://doi.org/10.17226/25353___.ZXV3Mjp1bml2ZXJzaXR5aG9zcGl0YWxzb3V0aGFtcHRvbjpjOm86MjhmNjE0OGY0ZTE0MTQ1MTk4OTQyZmMyNDE3NWI4MWI6Nzo2OWFiOmM5YWU5NGY5ZTE2N2M4MTRkNTVjYzc5MDIzNjk0MzdhNjQ5NzJiNjZmNDFiMWVkMGQxYTdiY2NkMDVlNzhiZjM6cDpGOk4)

^5^ National Health and Medical Research Council, Australian Government Department of Health and Ageing, New Zealand Ministry of Health. Nutrient Reference Values for Australia and New Zealand. Canberra: National Health and Medical Research Council; 2006, sodium updated 2017

**Supplementary Table 5: Randomized controlled trials in the general pediatric population**

| **Author; Year** | **Design** | **N** | **Country** | **methods** | **Outcomes reported** |
| --- | --- | --- | --- | --- | --- |
| Pomeranz, 2002 | Randomized controlled trial | 73 (age 0-6 mo) | Israel | Group 1: Formula diluted with low-sodium mineral water (LSMW) with sodium concentration of 32 mg/l (1.4 mmol/l).  Group 2: 33 infants whose formula was diluted with high-sodium mineral water (HSMW) with sodium concentration of 196 mg/l (8.5 mmol/l).  Group 3 (control group): 15 breastfed babies  After 8 weeks, group 1 reverted to a diet similar to that of group 2. | From the age of 6 weeks until week 8, mean arterial pressure (MAP), systolic blood pressure (SBP) and diastolic blood pressure (DBP) were found to be significantly greater in group 2 (HSTW). In parallel, the urinary sodium : creatinine ratio was significantly greater in this group. At week 24, blood pressure values in group 1 increased towards those of group 2. |
| Palacios, 2004 | Randomized crossover trial | 36 (22 black and 14 white girls, age 11-15 yrs) | USA | Na^+^ retention was assessed on a low sodium diet (1.3 g Na^+^/day, 57 mmol/day) and high sodium diet (4 g Na^+^/day, 172 mmol/day) during 3 weeks. | Mean daily Na^+^ retention was 357 ±69 mg (15.5 ±3.0 mmol) in blacks and 239 ±37 mg (10.4 ±1.6 mmol) in whites on the low-Na^+^ diet, and 991 ±138 mg (43.1 ±6.0 mmol) in blacks vs. 334 ±90 mg (14.5 ±3.9 mmol) in whites (p<0.001) on the high-Na^+^ diet. The greater Na^+^ retention in blacks was not accompanied by an increase in fecal or sweat Na^+^ excretion. BP and weight did not increase despite the Na+ retention |
| Cotter, 2013 | Randomized controlled trial | 139 (age 10-12 yrs) | Portugal | 6 months of three educational interventions in students from three classes each. Interventions consisted of: no additional action (controls), weekly lessons about the dangers of high salt intake (theoretical group) and both lessons and working practices in the school garden of planting, collection of herbs for salt substitution at home (practical group). | BP decreased by 8.2/6.5 mmHg in control group (n=31), by 3.8/0.6 mmHg in the theoretical group (n=43) and by 3.5/0.7 mmHg in practical group (n=53). Salt intake was reduced by 0.4 ±2.4 g/day in controls, 0.6 ±3.2 g/day in theoretical group and by 1.1 ±2.5 g/day in the practical group. |
| He, 2015 | Randomized controlled trial | 279 (age 9-11 yrs) | China | Children in the intervention group were educated on the harmful effects of salt and on how to reduce salt intake during the schools' usual health education lessons. Children then delivered the salt reduction message to their families. The intervention lasted for one school term (about 3.5 months). | Salt intake decreased in the intervention group, whereas it increased in the control group. The mean effect on salt intake for intervention vs. control group was -1.9 g/day (95% confidence interval: -2.6 to -1.3 g/day; p<0.001). The mean effect on systolic blood pressure was -0.8 mmHg (95%CI: -3.0 to 1.5 mm Hg; p=0.51) |
| He, 2022 | Randomized controlled trial | 592 healthy children | China | Children in the intervention group were taught, with support of an app, about salt reduction and assigned homework to encourage their families to participate in activities to reduce salt consumption. | Salt intake of the children increased both in the intervention and control groups, but to a lesser extent in the intervention group (mean effect of intervention after adjusting for confounding factors -0.25 g/day (95% CI: -0.61 to 0.12, p=0.18). Mean effect on SBP was -0.76 mmHg (-2.37 to 0.86, p=0.36) in children. |
| Bobowski, 2019 | Randomized controlled trial | 39 children; (ages 6-14 yrs, 67% females) |  | To determine whether 8-week exposure to low-sodium cereal (LSC) increased children's acceptance of its taste and changed their salty and sweet taste preferences. | Both treatment groups accepted and ate the assigned cereal throughout the 8-week exposure. Children showed no change in salt preference but readily ate the LSC for 8 consecutive weeks. Findings highlight the potential for reducing children's dietary salt intake by incorporating low-sodium foods in the home environment without more preferred higher-salt versions of these foods. |
| Ervina, 2021 | Randomized controlled trial | 148 children (mean age 11.9 ±0.3 years, 48% boys). | Norway | The objective of this study was to investigate the relationships between taste responsiveness and food liking in pre-adolescents. | Relationships between taste responsiveness and liking are product- and basic taste-dependent in addition to being subject-dependent. Strategies to improve acceptance by using sucrose as a suppressor for warning sensations of bitterness and sourness can be more or less effective depending on individual responsiveness to the basic tastes. |
| Toft, 2020 | Randomized controlled trial | 89 families (309 individuals) | Denmark | The aim was to examine the effects of two different salt reduction strategies on selected cardiovascular risk factors. The study was a four-month cluster randomised controlled study. Individuals were randomly assigned to either (A) gradually salt-reduced bread, (B) gradually salt-reduced bread and dietary counselling to further reduce salt intake and increase potassium intake or (C) standard bread (control). | Analysis showed a significant reduction in body fat percent (by -1.31%, 95% CI: -2.40 to -0.23) and a borderline significant reduction in total plasma cholesterol (-0.25 mmol/L, 95% CI: -0.51 to 0.01) and plasma renin (-0.19 pmol/L, 95% CI -0.39 to 0.00) in group A compared to the control group. Adjusted complete case analyses showed a significant reduction in total plasma cholesterol (-0.29 mmol/L, 95% CI : -0.50 to -0.08), plasma LDL cholesterol (-0.08 mmol/L, 95% CI: -0.15 to -0.00), plasma renin (-0.23 pmol/L, 95% CI: -0.41 to -0.05), plasma adrenaline (-0.03 nmol/L, 95% CI: -0.06 to -0.01)) and body fat percent (-1.53%, 95% CI: -2.51 to -0.54) in group A compared to the control group. No significant changes were found in group B compared to the control group.  In conclusion, receiving sodium reduced-bread was associated with beneficial changes in cardiovascular risk factors. No adverse effects were observed. |
| Yuan Ma, 2019 | Randomized controlled trial | 603 children (aged 10-12 yrs) | China | Investigation into whether social networks of children were associated with their behaviours to reduce salt intake.  A score was developed for assessing salt-reduction behaviours (SRB score) of children based on self-administered questionnaires. The SRB score was validated by the changes in salt intake measured by 24-hour urine collection in a random sample of 135 children. A 1-unit increase in SRB score was associated with a 0.31 g/day greater reduction in salt intake during the trial (95% CI : 0.06 to 0.57, p=0.016). | Children from families with more family members not supporting salt reduction had significantly lower SRB scores (p<0.0001). Children from a class with a smaller size and from a class with more friendship connections, as well as children having more friends within the class all showed higher SRB scores (all p<0.05). Children whose school teachers attended the intervention programme more frequently also had higher SRB scores (p=0.043). Social networks were associated with the behaviours to reduce salt intake in children. |
| Yasutake, 2019 | Randomized controlled trial | 100 university students | Japan | This study evaluated the use of a urinary salt excretion measurement device for salt-reduction education in a parallel randomized trial of two groups (51 controls, 49 self-monitoring). A survey with 24-hour home urine collection and BP measurement was conducted. Participants in the self-monitoring group measured their own urinary salt excretion level for 4 weeks, using the self-measurement device. | After intervention, 24-hour urinary sodium/potassium ratios showed no change in the control group (baseline score: 4.1 ±1.5; endline score: 4.2 ±2.0; p=0.723), but it decreased significantly in the self-monitoring group (baseline score: 4.0 ±1.7; endline score: 3.5 ±1.4; p=0.044). This change was significant even after adjusting for baseline and endline differences between groups using analysis of covariance (p=0.045).  The self-monitoring urinary salt excretion measurement device improved the 24-hour urinary sodium/potassium ratio. |
| Campbell, 2014 | Randomized controlled trial | 295 young children | Australia | This study aimed to describe food sources and demographic and behavioral correlates of sodium intake children using three unscheduled 24-hour recalls (when children were 9 and then 18 months of age) with mothers participating within an existing randomized controlled trial, the Melbourne Infant Feeding Activity and Nutrition Trial (InFANT) Program. | Mean (± standard deviation) Na intake was 486 ±232 mg at 9 months and had more than doubled to 1,069 mg ±331 mg) at 18 months of age. Fifty-four percent of children at 18 months exceeded the Recommended Daily Upper Level for Na intake, with bread, cheese, breakfast cereal, soup, and mixed dishes all important sources of Na at both ages. Yeast extracts, processed meats, and bread products became important additional sources at 18 months. A greater proportion of children in the highest Na-density tertile had ceased breastfeeding and had commenced solids at an earlier age. Key food sources of Na for children younger than 2 years are those that contribute to the whole population's high salt burden and highlight the essential role governments and food industry must play to reduce salt in commonly consumed foods. |
| Au, L;  2017 | Randomized controlled trial | 514  (257 in- person, 257 online) | USA | Study aim was to examine the impacts of traditional in-person and online nutrition education on changes in knowledge, self-efficacy, and behaviors related to reducing salt intake in low-income women enrolled in the The Special Supplemental Nutrition Program for Women, Infants, and Children.  Questionnaires assessing salt-related knowledge, self-efficacy, and behaviors were administered at baseline and 2 to 4 months and 9 months later from November 2014 through October 2015. | Positive changes in knowledge and self-efficacy were retained 2 to 4 months and 9 months later for both groups (p<0.05). Both groups reported significant changes in behaviors related to using less salt in cooking (p<0.0001) and eating fewer foods with salt added at the table or during cooking (p<0.001) at 2 to 4 months and 9 months. Both online and in-person education resulted in improvements during a 9-month period in knowledge, self-efficacy, and reported behaviors associated with reducing salt intake in a low-income population. |
| Valmorbida 2022 | Randomized controlled trial | Part of longitudinal study | Brazil | To examine effects of an early feeding intervention among low-income mothers on their children's energy and sodium intake and salt taste preferences at 12 years; and to identify age-related changes in dietary sodium sources. Mothers randomized to the intervention group received counseling on healthy eating practices for 1 year postpartum; the control group received no counseling. Two-day dietary recalls were obtained at 1 year (intervention end) and at 4-, 8-, and 12-year follow-up visits, from which foods were categorized as unprocessed, processed, or ultra-processed. At the 12-year visit, children's most preferred concentration of salt was measured using a validated, forced-choice, paired-comparison tracking method, and pubertal stage was self-assessed. | The intervention group had reduced energy intake compared with controls in all food categories at 1 year (p=0.04) but not at the other time points. Sodium intake from processed foods increased from 4 to 12 years and from ultra-processed foods from 1 to 4; intake from unprocessed foods decreased from 1 to 8 year (all p<0.01). At 12 years, children in early stages of puberty (Tanner stages 1-3; p=0.04) or in the ≥75th percentile of sodium intake (p<0.01) preferred significantly higher concentrations of salt than the other children. |
| Singal 2001 | RCT (long term outcomes of previous RCT by Lucas 1988) | 216 preterm children (age 13-16 yrs) | UK | Dietary interventions at birth were: donated banked breastmilk vs. preterm (high sodium) formula (n=42) and standard term (low sodium) formula (n=44) vs. preterm formula. | Mean arterial BP at age 13-16 years was lower in the 66 children assigned banked breastmilk (alone or in addition to mother's milk) (low sodium) than in the 64 assigned preterm formula (high sodium) (mean ±standard deviation: 81.9 ±7.8 vs. 86.1 ±6.5 mmHg.  In non-randomised analyses, the proportion of enteral intake as human milk in the neonatal period was inversely related to later mean arterial pressure (beta: -0.3 mmHg per 10% increase (95% CI: -0.5 to -0.1); p=0.006)  No differences were found in the term formula vs. preterm formula comparison |
| Hartnoll 2000 | RCT | 24 infants (age 25-30 weeks' gestation) | UK | Preterm infants were stratified and randomly assigned according to gender and gestational age, to receive a sodium intake of 4 mmol/kg/day beginning either on the second day after birth or when weight loss of 6% of birthweight had been achieved. | In the delayed sodium supplementation group, but not the early group, there was a significant loss of total body water during the first week (delayed -44 ml/kg, p=0.048; early 6 ml/kg, p=0.970).  By day 14 the delayed, but not the early group, also had a significant reduction in extracellular fluid volume (delayed -53 ml/kg, p=0.01; early -37 ml/kg, p=0.2)  Significant alteration in body composition at the end of the first week (total body weight: delayed 791 ml/kg; early 849 ml/kg, p=0.013). By day 14 there were once again no significant differences in body composition between the two groups |

**Abbreviations**

95% CI: 95% confidence interval

BP: blood pressure

DBP: diastolic blood pressure

HSTW: high-salt tap water

LSC : low-sodium cereal

LSTW: low-salt tap water

MAP: mean arterial pressure

SBP: systolic blood pressure

SRB: salt-reduction behaviours

**Supplementary Figure 2. Examples of front-of-pack labelling**

**Supplementary Table 6: Instructions to prepare of a sodium chloride solution at home**

| 1 | Preliminary note: extreme caution should be used when preparing this kind of solution |
| --- | --- |
| 2 | Consider the sodium (Na) content of table salt (NaCl)  1 g Na = 44 mEq Na = 44 mmol Na  1 g NaCl = 400 mg Na = 17 mEq NaCl = 17 mmol NaCl  5 g NaCl = 2 g Na = 85 mEq Na = 85 mmol Na  5.9 g NaCl= 2.3 g Na = 100 mEq Na = 100 mmol Na |
| 3 | Add a specific amount of table salt to a measured amount of water  Example, to prepare a 1 mEq/ml (1 mmol/ml) sodium solution, add 1 level teaspoon of table salt to 100 ml water:  1 ml solution = 1 mEq Na = 1 mmol Na  1 ml solution = 23 mg Na  100 ml solution = 2300 mg Na  100 ml solution = 5.9 g table salt (40% Na)  100 ml solution = 1 level teaspoon table salt (6 g) |
| 4 | Administer the prescribed amount orally or by adding to the enteral feed |
| 5 | Store in refrigerator and discard after 2 weeks at latest |

**APPENDIX A- Relevant studies about sodium**

General papers on sodium kinetics

- Canaud B, Kooman J, Selby NM, Taal M, Francis S, Kopperschmidt P, Maierhofer A, Kotanko P, Titze J (2019) Sodium and water handling during hemodialysis: new pathophysiologic insights and management approaches for improving outcomes in end-stage kidney disease. Kidney Int 95:296-309.
- Dahlmann A, Dorfelt K, Eicher F, Linz P, Kopp C, Mossinger I, Horn S, Buschges-Seraphin B, Wabel P, Hammon M, Cavallaro A, Eckardt KU, Kotanko P, Levin NW, Johannes B, Uder M, Luft FC, Muller DN, Titze JM (2015) Magnetic resonance-determined sodium removal from tissue stores in hemodialysis patients. Kidney Int 87:434-441.
- Schneider MP, Raff U, Kopp C, Scheppach JB, Toncar S, Wanner C, Schlieper G, Saritas T, Floege J, Schmid M, Birukov A, Dahlmann A, Linz P, Janka R, Uder M, Schmieder RE, Titze JM, Eckardt KU (2017) Skin Sodium Concentration Correlates with Left Ventricular Hypertrophy in CKD. J Am Soc Nephrol 28:1867-1876.
- Olde Engberink RHG, Selvarajah V, Vogt L (2020) Clinical impact of tissue sodium storage. Pediatr Nephrol 35:1373-1380.
- Twardowski ZJ (2008) Sodium, hypertension, and an explanation of the "lag phenomenon" in hemodialysis patients. Hemodial Int 12:412-425.
- Deger SM, Wang P, Fissell R, Ellis CD, Booker C, Sha F, Morse JL, Stewart TG, Gore JC, Siew ED, Titze J, Ikizler TA (2017) Tissue sodium accumulation and peripheral insulin sensitivity in maintenance hemodialysis patients. J Cachexia Sarcopenia Muscle 8:500-507.
- Sahinoz M, Tintara S, Deger SM, Alsouqi A, Crescenzi RL, Mambungu C, Vincz A, Mason O, Prigmore HL, Guide A, Stewart TG, Harrison D, Luft FC, Titze J, Alp Ikizler T (2020) Tissue sodium stores in peritoneal dialysis and hemodialysis patients determined by 23-sodium magnetic resonance imaging. Nephrol Dial Transplant 36:1307-1317.
- Friedrich AC, Linz P, Nagel AM, Rosenhauer D, Horn S, Schiffer M, Uder M, Kopp C, Dahlmann A (2022) Hemodialysis Patients with Cardiovascular Disease Reveal Increased Tissue Na+ Deposition. Kidney Blood Press Res 47:185-193.
- Salerno FR, Akbari A, Lemoine S, Filler G, Scholl TJ, McIntyre CW (2022) Outcomes and predictors of skin sodium concentration in dialysis patients. Clin Kidney J 15:1129-1136.

Sources of dietary sodium

Andersen L, Rasmussen LB, Larsen EH and Jakobsen J (2009) Intake of household salt in a Danish population. European Journal of Clinical Nutrition, 63, 598–604

Bailey RL, Catellier DJ, Jun S, Dwyer JT, Jacquier EF, Anater AS, Eldridge AL. (2018) Total Usual Nutrient Intakes of US Children (Under 48 Months): Findings from the Feeding Infants and Toddlers Study (FITS) J Nutr. 148(9S):1557S-1566S

Banks D, Birke M, Flem B and Reimann C (2015) Inorganic chemical quality of European tap-water: 1. Distribution of parameters and regulatory compliance. Applied Geochemistry, 59, 200–210.

Bhat S, Marklund M, Henry ME, Appel LJ, Croft KD, Neal B, Wu JHY (2020) A Systematic Review of the Sources of Dietary Salt Around the World. Adv Nutr. 11(3):677-686

Birch LL, Doub AE. (2014) Learning to eat: birth to age 2 y. Am J Clin Nutr 99:723S–8S.

Butte NF, Fox MK, Briefel RR, Siega-Riz AM, Wwyer J, Deming DM, Reidy K (2010) Nutrient Intakes of US Infants, Toddlers, and Preschoolers Meet or Exceed Dietary Reference Intakes. Supplement to the Journal of the AMERICAN DIETETIC ASSOCIATION

Campbell KJ, Hendrie G, Nowson C, Grimes CA, Riley M, Lioret S, McNaughton S. (2014) Sources and correlates of sodium consumption in the first 2 years of life. J Acad Nutr Diet. 114:1525-1532.

Commission Directive 2006/125/EC of 5 December 2006 on processed cereal-based foods and baby foods for infants and young children. OJ L 339, 6.12.2006, p. 20

Commission Delegated Regulation (EU) 2016/127 of 25 September 2015 supplementing Regulation (EU) No 609/2013 of the European Parliament and of the Council as regards the specific compositional and information requirements for infant formula and follow-on formula and as regards requirements on information relating to infant and young child feeding

Devaney B, Ziegler P, Pac S, Karwe V, Barr SI (2004) Nutrient Intakes of Infants and Toddlers. Journal of THE AMERICAN DIETETIC ASSOCIATION Suppl 1 Volume 104 Number 1

Elmadfa I, European nutrition and health report, Forum of Nutrition vol. 622009. (Web. 2 June 2014. <http://www.univie.ac.at/enhr/downloads/enhrii_book.pdf>.).

European Commission, 2012. Survey on Members States ’implementation of the EU salt reduction framework.26 pp. Available online:https://ec.europa.eu/health//sites/health/files/nutrition_physical_activity/docs/salt_report1_en.pdf

European Commission Directorate-General Health and Consumers, Survey on members states' implementation of the EU salt reduction framework(Web. 5 June 2014.) [http://ec.europa.eu/health/nutrition_physical_activity/docs/salt_report1_en.pdf 2013](http://ec.europa.eu/health/nutrition_physical_activity/docs/salt_report1_en.pdf%202013).

Grimes CA, Wright JD, Liu K, Nowson CA, Loria CM. (2013) Dietary sodium intake is associated with total fluid and sugar-sweetened beverage consumption in US children and adolescents aged 2–18 y: NHANES 2005–2008. Am J Clin Nutr 98:189–96.

Guallar-Castillón P, Muñoz-Pareja M, Aguilera MT, León-Muñoz LM, Rodríguez-Artalejo F (2013) Food sources of sodium, saturated fat and added sugar in the Spanish hypertensive and diabetic population. Atherosclerosis. 229(1):198-205

Harnack LJ, Cogswell ME, Shikany JM, et al. (2017) Sources of sodium in US adults from 3 geographic regions. Circulation 135:1775–83

James WP, Ralph A, Sanchez-Castillo CP (1987) The dominance of salt in manufactured food in the sodium intake of affluent societies. Lancet. 1:426–429

Kastorini C, Panagiotakos D (2009) Dietary patterns and prevention of type 2 diabetes: from research to clinical practice; a systematic review, Curr. Diabetes Rev. 5(4) 221–227

Leclercq C and Ferro-Luzzi A (1991) Total and domestic consumption of salt and their determinants in three regions of Italy. European Journal of Clinical Nutrition 45, 151–159

Maalouf J, Cogswell ME, Yuan K, Martin C, Gunn JP, Pehrsson P, Merritt R, Bowman B (2015) Top sources of dietary sodium from birth to age 24 mo, United States, 2003-2010. Am J Clin Nutr. 101(5):1021-8

Melhuish, E., Lindeback, R. & Lambert, K (2022) Scoping review of the dietary intake of children with chronic kidney disease. Pediatr Nephrol 37, 1995–2012

Meneton P, Lafay L, Tard A, Dufour A, Ireland J, Ménard J, Volatier JL (2009) Dietary sources and correlates of sodium and potassium intakes in the French general population. Eur J Clin Nutr. 63(10):1169-75

Monteiro, C.A., Cannon, G., Lawrence, M., Costa Louzada, M.L, Pereira Machado P (2019) Ultra-processed foods, diet quality, and health using the NOVA classification system. Rome, FAO

Reinivuo H, Valsta LM, Laatikainen T, Tuomilehto J, Pietinen P. (2006) Sodium in the Finnish diet: II trends in dietary sodium intake and comparison between intake and 24-h excretion of sodium. Eur J Clin Nutr. 60(10):1160-7

Rossum van CTM. The diet of the Dutch Results of the Dutch National Food Consumption Survey 2019-2021 on food consumption and evaluation with dietary guidelines. RIVM rapport 2022-0190 <https://www.rivm.nl/bibliotheek/rapporten/2022-0190.pdf>

Scourboutakos MJ., L’Abbé MR. (2013). Sodium Levels in Canadian fast-food and sit-down restaurants. Can J Public Health. 104(1):e2–e8.

Stimec M, Kobe H, Smole K, Kotnik P, Sirca-Campa A, Zupancic M, Battelino T, Krzisnik C, Fidler Mis N (2009) Adequate iodine intake of Slovenian adolescents is primarily attributed to excessive salt intake. Nutr Res. 29(12):888-96

Tedstone A. Public Health England. Salt targets 2017: Progress report. A report on the food industry’s progress towards meeting the 2017 salt targets. December 2018. [Available from: [www.gov.uk/government/publications](http://www.gov.uk/government/publications)]

Zhou BF, Stamler J, Dennis B, Moag-Stahlberg A, Okuda N, Robertson C, Zhao L, Chan Q, Elliott P, INTERMAP Research Group (2003) Nutrient intakes of middle-aged men and women in China, Japan, United Kingdom, and United States in the late 1990s: The INTERMAP study. J Hum Hypertens. 17:623–630.

Cuadrado-Soto E, Peral-Suarez Á, Aparicio A, Perea JM, Ortega RM, López-Sobaler AM. (2018) Sources of Dietary Sodium in Food and Beverages Consumed by Spanish Schoolchildren between 7 and 11 Years Old by the Degree of Processing and the Nutritional Profile. Nutrients 10(12):1880.

Teixeira AZA (2018) Sodium content and food additives in major brands of Brazilian children's foods. Cien Saude Colet 23(12):4065-4075

Carrigan A, Klinger A, Choquette SS, Luzuriaga-McPherson A, Bell EK, Darnell B, Gutiérrez OM (2014) Contribution of food additives to sodium and phosphorus content of diets rich in processed foods. J Ren Nutr. 24(1):13-9

Azoulay A, Garzon P and Eisenberg MJ (2001) Comparison of the mineral content of tap water and bottled waters. Journal of General Internal Medicine 16: 168–175.

- Magriplis E, Farajian P, Pounis GD, Risvas G, Panagiotakos DB, Zampelas A (2011) High sodium intake of children through 'hidden' food sources and its association with the Mediterranean diet: the GRECO study. J Hypertens. 29(6):1069-76
- Sanchez-Castillo CP, Warrender S, Whitehead TP, James WP (1986) An assessment of the sources of dietary salt in a British population. Clin Sci (Lond). 72:95–102.

Dietary sodium assessment

- Bentley B (2006) A review of methods to measure dietary sodium intake. J Cardiovasc Nurs. 21(1):63-7
- Rhodes DG, Murayi T, Clemens JC, Baer DJ, Sebastian RS, Moshfegh AJ (2013) The USDA Automated Multiple-Pass Method accurately assesses population sodium intakes. Am J Clin Nutr. 97(5):958-64
- Espeland MA, Kumanyika S, Wilson AC, Reboussin DM, Easter L, Self M, Robertson J, Brown WM, McFarlane M; TONE Cooperative Research Group (2001) Statistical issues in analyzing 24-hour dietary recall and 24-hour urine collection data for sodium and potassium intakes. Am J Epidemiol. 153(10):996-1006
- Mark A Espeland, Shiriki Kumanyika, Alan C Wilson, Sara Wilcox, Dinnie Chao, Judy Bahnson, David M Reboussin, Linda Easter, Beiyao Zheng (2001) Lifestyle Interventions Influence Relative Errors in Self-Reported Diet Intake of Sodium and Potassium, Annals of Epidemiology 11: 2001
- Gallani MC, Proulx-Belhumeur A, Almeras N, Després JP, Doré M, Giguère JF (2020) Development and Validation of a Salt Food Frequency Questionnaire (FFQ-Na) and a Discretionary Salt Questionnaire (DSQ) for the Evaluation of Salt Intake among French-Canadian Population. Nutrients. 13(1):105

Urinary sodium

- Campino C, Hill C, Baudrand R, Martinez-Aguayo A, Aglony M, Carrasco CA, Ferrada C, Loureiro C, Vecchiola A, Bancalari R, Grob F, Carvajal CA, Lagos CF, Valdivia C, Tapia-Castillo A, Fuentes CA, Mendoza C, Garcia H, Uauy R, Fardella CE (2016) Usefulness and Pitfalls in Sodium Intake Estimation: Comparison of Dietary Assessment and Urinary Excretion in Chilean Children and Adults. Am J Hypertens 29:1212-1217.

Plasma sodium

- He FJ, Fan S, Macgregor GA, Yaqoob MM (2013) Plasma sodium and blood pressure in individuals on haemodialysis. J Hum Hypertens. 27(2):85-9
- Suckling RJ, Swift PA, He FJ, Markandu ND, MacGregor GA (2013) Altering plasma sodium concentration rapidly changes blood pressure during haemodialysis. Nephrol Dial Transplant 28(8):2181-6

Sodium in medications

- Nakayama Y, Ueda K, Yamagishi SI, Sugiyama M, Yoshida C, Kurokawa Y, Nakamura N, Moriyama T, Kodama G, Minezaki T, Ito S, Nagata A, Taguchi K, Yano J, Kaida Y, Shibatomi K, Fukami K (2018) Compared effects of calcium and sodium polystyrene sulfonate on mineral and bone metabolism and volume overload in pre-dialysis patients with hyperkalemia. Clin Exp Nephrol 22:35-44
- Le Palma K, Pavlick ER, Copelovitch L (2018) Pretreatment of enteral nutrition with sodium polystyrene sulfonate: effective, but beware the high prevalence of electrolyte derangements in clinical practice. Clin Kid J 11: 166-71
- Zeng C, Rosenberg L, Li X, Djousse L, Wei J, Lei G, Zhang Y (2022) Sodium-containing acetaminophen and cardiovascular outcomes in individuals with and without hypertension. Eur Heart J 43(18):1743-1755
- Ubeda A, Llopico J, Sanchez MT (2009) Blood pressure reduction in hypertensive patients after withdrawal of effervescent medication. Pharmacoepidemiol Drug Saf 18(5):417-9
- George J, Majeed W, Mackenzie IS, Macdonald TM, Wei L (2013) Association between cardiovascular events and sodium-containing effervescent, dispersible, and soluble drugs: nested case-control study. BMJ. 347:f6954
- Wei L, Mackenzie IS, MacDonald TM, George J (2014) Cardiovascular risk associated with sodium-containing medicines. Expert Opin Drug Saf. 13(11):1515-23
- Benitez-Camps M, Morros Padrós R, Pera-Pujadas H, Dalfó Baqué A, Bayó Llibre J, Rebagliato Nadal O, Cortès Martinez J, García Sangenís A, Roca Saumell C, Coll de Tuero G, Vinyoles-Bargalló E; in representation of Paracetamol Investigators (2018) Effect of effervescent paracetamol on blood pressure: a crossover randomized clinical trial. J Hypertens 36(8):1656-1662

Diuretics

- Zamboli P, De Nicola L, Minutolo R, Chiodini P, Crivaro M, Tassinario S, Bellizzi V, Conte G (2011) Effect of furosemide on left ventricular mass in non-dialysis chronic kidney disease patients: a randomized controlled trial. Nephrol Dial Transplant. 26(5):1575-83
- Sibbel S, Walker AG, Colson C, Tentori F, Brunelli SM, Flythe J (2019) Association of Continuation of Loop Diuretics at Hemodialysis Initiation with Clinical Outcomes. Clin J Am Soc Nephrol 14:95-102.
- Saran R, Padilla RL, Gillespie BW, Heung M, Hummel SL, Derebail VK, Pitt B, Levin NW, Zhu F, Abbas SR, Liu L, Kotanko P, Klemmer P (2017) A Randomized Crossover Trial of Dietary Sodium Restriction in Stage 3-4 CKD. Clin J Am Soc Nephrol 12 (3):399-407
- van Olden RW, Guchelaar HJ, Struijk DG, Krediet RT, Arisz L (2003) Acute effects of high-dose furosemide on residual renal function in CAPD patients. Perit Dial Int 23 (4):339-347
- Medcalf JF, Harris KP, Walls J (2001) Role of diuretics in the preservation of residual renal function in patients on continuous ambulatory peritoneal dialysis. Kidney international 59 (3):1128-1133
- Bragg-Gresham JL, Fissell RB, Mason NA, Bailie GR, Gillespie BW, Wizemann V, Cruz JM, Akiba T, Kurokawa K, Ramirez S, Young EW (2007) Diuretic use, residual renal function, and mortality among hemodialysis patients in the Dialysis Outcomes and Practice Pattern Study (DOPPS). American journal of kidney diseases 49 (3):426-431
- Vasavada N, Saha C, Agarwal R (2003) A double-blind randomized crossover trial of two loop diuretics in chronic kidney disease. Kidney Int. 64(2):632-40
- Bovée DM, Visser WJ, Middel I, De Mik-van Egmond A, Greupink R, Masereeuw R, Russel FGM, Danser AHJ, Zietse R, Hoorn EJ. (2020) A Randomized Trial of Distal Diuretics versus Dietary Sodium Restriction for Hypertension in Chronic Kidney Disease. J Am Soc Nephrol. 31(3):650-662.
- Teles F, Peçanha de Miranda Coelho JA, Albino RM, Verçosa Pacheco FC, Rodrigues de Oliveira E, Silveira MAD, Diógenes M Feitosa A, Bezerra R (2023) Effectiveness of thiazide and thiazide-like diuretics in advanced chronic kidney disease: a systematic review and meta-analysis. Ren Fail. 45(1):2163903.

Dialysis and sodium

- Borzych-Dużałka D SR, Ranchin B, Zhai Y, Paglialonga F, Kari J, Han Ahn Y, Subhi Awad H, Loza R, Hooman N, Ericson R, Drożdz D, Kaur A, Bakkaloglu SA, Samaille C, Lee M, Tellier S, Warady BA, Schaefer F, Schmitt CP (2023) Prospective study of modifiable risk factors of arterial hypertension and left ventricular hypertrophy in pediatric hemodialysis patients. Kidney Int Rep. 9(6):1694-1704.
- Song JH, Lee SW, Suh CK, Kim MJ (2002) Time-averaged concentration of dialysate sodium relates with sodium load and interdialytic weight gain during sodium-profiling hemodialysis. Am J Kidney Dis 40:291-301.
- Basile C, Pisano A, Lisi P, Rossi L, Lomonte C, Bolignano D (2016) High versus low dialysate sodium concentration in chronic haemodialysis patients: a systematic review of 23 studies. Nephrol Dial Transplant 31 (4):548-563
- Flythe JE, Mc Causland FR (2017) Dialysate Sodium: Rationale for Evolution over Time. Semin Dial 30:99-111.
- Gul A, Miskulin DC, Paine SS, Narsipur SS, Arbeit LA, Harford AM, Weiner DE, Schrader R, Horowitz BL, Zager PG (2016) Comparison of Prescribed and Measured Dialysate Sodium: A Quality Improvement Project. Am J Kidney Dis 67:439-445.
- Stragier A, Lopot F, Svara F, Polakovic V (2018) Fallacies and Pitfalls of Dialysis Sodium Prescription and Control. Blood Purif 46:27-33.
- Shendi AM, Davenport A (2021) The difference between delivered and prescribed dialysate sodium in haemodialysis machines. Clin Kidney J 14:863-868.
- Ng JK, Smyth B, Marshall MR, Kang A, Pinter J, Bassi A, Krishnasamy R, Rossignol P, Rocco MV, Li Z, Jha V, Hawley CM, Kerr PG, GL DIT, Woodward M, Jardine AM, Committee RS (2021) Relationship between measured and prescribed dialysate sodium in haemodialysis: a systematic review and meta-analysis. Nephrol Dial Transplant 36:695-703.
- Dunlop JL, Vandal AC, Marshall MR (2019) Low dialysate sodium levels for chronic haemodialysis. Cochrane Database Syst Rev 1:CD011204.
- Marshall MR, Vandal AC, de Zoysa JR, Gabriel RS, Haloob IA, Hood CJ, Irvine JH, Matheson PJ, McGregor DOR, Rabindranath KS, Schollum JBW, Semple DJ, Xie Z, Ma TM, Sisk R, Dunlop JL (2020) Effect of Low-Sodium versus Conventional Sodium Dialysate on Left Ventricular Mass in Home and Self-Care Satellite Facility Hemodialysis Patients: A Randomized Clinical Trial. J Am Soc Nephrol 31:1078-1091.
- Geng X, Song Y, Hou B, Ma Y, Wang Y (2020) The efficacy and safety of low dialysate sodium levels for patients with maintenance haemodialysis: A systematic review and meta-analysis. Int J Surg 79:332-339.
- Marsenic O, Anderson M, Couloures KG, Hong WS, Kevin Hall E, Dahl N (2016) Effect of the decrease in dialysate sodium in pediatric patients on chronic hemodialysis. Hemodial Int 20:277-285.
- Caporale O, Consolo S, Grassi FS, Grassi MR, Puccio G, Montini G, Paglialonga F (2022) Low dialysate sodium in children and young adults on maintenance hemodialysis: a prospective, randomized, crossover study. Pediatr Nephrol 38: 1599-1607
- Causland FRM, Ravi KS, Curtis KA, Kibbelaar ZA, Short SAP, Singh AT, Correa S, Waikar SS (2022) A randomized controlled trial of two dialysate sodium concentrations in hospitalized hemodialysis patients. Nephrol Dial Transplant 37:1340-1347.
- Meira FS, Poli de Figueiredo CE, Figueiredo AE (2007) Influence of sodium profile in preventing complications during hemodialysis. Hemodial Int 11 Suppl 3:S29-32.
- Ponce P, Pinto B, Wojke R, Maierhofer AP, Gauly A (2020) Evaluation of intradialytic sodium shifts during sodium controlled hemodialysis. Int J Artif Organs 43:620-624.
- Kuhlmann U, Maierhofer A, Canaud B, Hoyer J, Gross M (2019) Zero Diffusive Sodium Balance in Hemodialysis Provided by an Algorithm-Based Electrolyte Balancing Controller: A Proof of Principle Clinical Study. Artif Organs 43:150-158.
- Sagova M, Wojke R, Maierhofer A, Gross M, Canaud B, Gauly A (2019) Automated individualization of dialysate sodium concentration reduces intradialytic plasma sodium changes in hemodialysis. Artif Organs 43:1002-1013.
- Thein H, Haloob I, Marshall MR (2007) Associations of a facility level decrease in dialysate sodium concentration with blood pressure and interdialytic weight gain. Nephrol Dial Transplant 22: 2630-2639
- Munoz Mendoza J, Arramreddy R, Schiller B (2015) Dialysate sodium: choosing the optimal hemodialysis bath. Am J Kidney Dis 66: 710-720
- Wong MM, McCullough KP, Bieber BA, Bommer J, Hecking M, Levin NW, McClellan WM, Pisoni RL, Saran R, Tentori F, Tomo T, Port FK, Robinson BM (2017) Interdialytic Weight Gain: Trends, Predictors, and Associated Outcomes in the International Dialysis Outcomes and Practice Patterns Study (DOPPS). Am J Kidney Dis. 69(3):367-379
- Fischbach M, Schmitt CP, Shroff R, Zaloszyc A, Warady BA (2016) Increasing sodium removal on peritoneal dialysis: applying dialysis mechanics to the peritoneal dialysis prescription. Kidney Int 89:761-766.
- Wang T, Waniewski J, Heimburger O, Werynski A, Lindholm B (1997) A quantitative analysis of sodium transport and removal during peritoneal dialysis. Kidney Int 52:1609-1616.
- Borrelli S, La Milia V, De Nicola L, Cabiddu G, Russo R, Provenzano M, Minutolo R, Conte G, Garofalo C, Study group Peritoneal Dialysis of Italian Society of N (2019) Sodium removal by peritoneal dialysis: a systematic review and meta-analysis. J Nephrol 32:231-239.
- Fischbach M, Zaloszyc A, Schaefer B, Schmitt CP (2017) Should sodium removal in peritoneal dialysis be estimated from the ultrafiltration volume? Pediatr Nephrol 32:419-424.
- Davies S, Carlsson O, Simonsen O, Johansson AC, Venturoli D, Ledebo I, Wieslander A, Chan C, Rippe B (2009) The effects of low-sodium peritoneal dialysis fluids on blood pressure, thirst and volume status. Nephrol Dial Transplant 24:1609-1617.
- Davies S, Haraldsson B, Vrtovsnik F, Schwenger V, Fan S, Klein A, Atiye S, Gauly A (2020) Single-dwell treatment with a low-sodium solution in hypertensive peritoneal dialysis patients. Perit Dial Int 40:446-454.
- Rutkowski B, Tam P, van der Sande FM, Vychytil A, Schwenger V, Himmele R, Gauly A, Low Sodium Balance Study G (2016) Low-Sodium Versus Standard-Sodium Peritoneal Dialysis Solution in Hypertensive Patients: A Randomized Controlled Trial. Am J Kidney Dis 67:753-761.
- Rutkowski B, Tam P, van der Sande FM, Vychytil A, Schwenger V, Klein G, Himmele R, Gauly A, Low Sodium balance Study G (2019) Residual Renal Function and Effect of Low-Sodium Solution on Blood Pressure in Peritoneal Dialysis Patients. Perit Dial Int 39:335-343.
- Rao VS, Turner JM, Griffin M, Mahoney D, Asher J, Jeon S, Yoo PS, Boutagy N, Feher A, Sinusas A, Wilson FP, Finkelstein F, Testani JM (2020) First-in-Human Experience With Peritoneal Direct Sodium Removal Using a Zero-Sodium Solution: A New Candidate Therapy for Volume Overload. Circulation 141:1043-1053.

Patients with increased sodium losses

- Fine BP, Ty A, Lestrange N, Maher E, Levine OR (1987) Diuretic induced growth failure in rats and its reversal by sodium repletion. J Pharmacol Exp Ther 242: 85–89
- Wassner S (1989) Altered growth and protein turnover in rats fed sodium-deficient diets. Pediatr Res 26: 608–613
- Fine BP, Ty A, Lestrange N, Levine R (1987) Sodium deprivation growth failure in the rat: Alterations in tissue composition and fluid spaces. J Nutr 117: 1623–1628,
- Declercq D, Van Braeckel E, Marchand S, Van Daele S, Van Biervliet S (2020) Sodium Status and Replacement in Children and Adults Living with Cystic Fibrosis: A Narrative Review. J Acad Nutr Diet. 120(9):1517-1529
- Scurati-Manzoni E, Fossali EF, Agostoni C, Riva E, Simonetti GD, Zanolari-Calderari M, Bianchetti MG, Lava SA (2014) Electrolyte abnormalities in cystic fibrosis: systematic review of the literature. Pediatr Nephrol. 29(6):1015-23
- Mansour F, Petersen D, De Coppi P, Eaton S. (2014) Effect of sodium deficiency on growth of surgical infants: a retrospective observational study. Pediatr Surg Int. 30(12):1279-84
- Trautmann T, Bang C, Franke A, Vincent D, Reinshagen K, Boettcher M (2020) The Impact of Oral Sodium Chloride Supplementation on Thrive and the Intestinal Microbiome in Neonates With Small Bowel Ostomies: A Prospective Cohort Study. Front Immunol. 10;11:1421
- Schwarz KB, Ternberg JL, Bell MJ, Keating JP (1983) Sodium needs of infants and children with ileostomy. J Pediatr. 102(4):509-13

Recommended levels in healthy children

- Health Council of the Netherlands. Guidelines for a healthy diet 2006. The Hague: Health Council of the Netherlands, 2006; publication no. 2006/21E, updated in 2015.
- Kromhout D, Spaaij CJ, de Goede J, Weggemans RM. The 2015 Dutch food-based dietary guidelines. Eur J Clin Nutr. 2016 Aug;70(8):869-78. doi: 10.1038/ejcn.2016.52. Epub 2016 Apr 6. PMID: 27049034; PMCID: PMC5399142
- Deutsche Gesellschaft für Ernährung, Österreichische Gesellschaft für Ernährung, Schweizerische Gesellschaft für Ernährung, 2015. Referenzwerte für die Nährstoffzufuhr. 2. Auflage, 1. Ausgabe. DGE, Bonn, Germany
- Department of Health. Dietary reference values for food energy and nutrients for the United Kingdom. Report of the Panel on Dietary Reference Values of the Committee on Medical Aspects of Food Policy. HMSO, London, UK, 1991.

Salt sensitivity

- Lucas A, Morley R, Hudson GJ, Bamford MF, Boon A, Crowle P, Dossetor JF, Pearse R (1988) Early sodium intake and later blood pressure in preterm infants. Arch Dis Child 63(6):656-7
- Singhal A, Cole TJ, Lucas A (2001) Early nutrition in preterm infants and later blood pressure: two cohorts after randomised trials. Lancet 357(9254):413-9
- Hartnoll G, Bétrémieux P, Modi N (2000) Randomised controlled trial of postnatal sodium supplementation on body composition in 25 to 30 week gestational age infants. Arch Dis Child Fetal Neonatal Ed. 82(1):F24-8
- Geleijnse JM, Hofman A, Witteman JC, Hazebroek AA, Valkenburg HA, Grobbee DE (1997) Long-term effects of neonatal sodium restriction on blood pressure. Hypertension. 29(4):913-7
- Ruys CA, Rotteveel J, van de Lagemaat M, Lafeber HN, Finken MJJ (2018) Salt sensitivity of blood pressure at age 8 years in children born preterm. J Hum Hypertens.32(5):367-376
- He FJ, Marrero NM, MacGregor GA (2008) Salt intake is related to soft drink consumption in children and adolescents: a link to obesity? Hypertension. 51(3):629-34
- Grimes CA, Riddell LJ, Campbell KJ, Nowson CA (2013) Dietary salt intake, sugar-sweetened beverage consumption, and obesity risk. Pediatrics. 131(1):14-21
- Correia-Costa L, Cosme D, Nogueira-Silva L, Morato M, Sousa T, Moura C, Mota C, Guerra A, Albino-Teixeira A, Areias JC, Schaefer F, Lopes C, Afonso AC, Azevedo A. (2016) Gender and obesity modify the impact of salt intake on blood pressure in children. Pediatr Nephrol. 31(2):279-88
- Grimes CA, Bolton KA, Booth AB, Khokhar D, Service C, He FH, Nowson CA (2021) The association between dietary sodium intake, adiposity and sugar-sweetened beverages in children and adults: a systematic review and meta-analysis. Br J Nutr 126(3):409-427
- Lava SA, Bianchetti MG, Simonetti GD (2015) Salt intake in children and its consequences on blood pressure. Pediatr Nephrol. 30(9):1389-96
- Elijovich F, Weinberger MH, Anderson CA, Appel LJ, Bursztyn M, Cook NR, Dart RA, Newton-Cheh CH, Sacks FM, Laffer CL; American Heart Association Professional and Public Education Committee of the Council on Hypertension; Council on Functional Genomics and Translational Biology; and Stroke Council (2016) Salt Sensitivity of Blood Pressure: A Scientific Statement From the American Heart Association. Hypertension. 68(3):e7-e46

Dietary sodium and blood pressure

- Rios-Leyvraz M, Bloetzer C, Chatelan A, Bochud M, Burnier M, Santschi V, Paradis G, Tabin R, Bovet P, Chiolero A (2019) Sodium intake and blood pressure in children with clinical conditions: A systematic review with meta-analysis. J Clin Hypertens (Greenwich).;21(1):118-126
- Sacks FM, Svetkey LP, Vollmer WM, Appel LJ, Bray GA, Harsha D, Obarzanek E, Conlin PR, Miller ER 3rd, Simons-Morton DG, Karanja N, Lin PH; DASH-Sodium Collaborative Research Group (2001) Effects on blood pressure of reduced dietary sodium and the Dietary Approaches to Stop Hypertension (DASH) diet. DASH-Sodium Collaborative Research Group.N Engl J Med. 4;344(1):3-10
- Meuleman Y, Hoekstra T, Dekker FW, Navis G, Vogt L, van der Boog PJM, Bos WJW, van Montfrans GA, van Dijk S; ESMO Study Group (2017) Sodium Restriction in Patients With CKD: A Randomized Controlled Trial of Self-management Support. Am J Kidney Dis. 69(5):576-586 -
- Wise PM, Hansen JL, Reed DR, Breslin PA (2007) Twin study of the heritability of recognition thresholds for sour and salty taste. Chem Senses 32: 749-754
- Tomson CR (2001) Advising dialysis patients to restrict fluid intake without restricting sodium intake is not based on evidence and is a waste of time. Nephrol Dial Transplant 16:1538-1542.
- Ellison DH, Welling P (2021) Insights into Salt Handling and Blood Pressure. N Engl J Med 385:1981-1993.
- Kayikcioglu M, Tumuklu M, Ozkahya M, Ozdogan O, Asci G, Duman S, Toz H, Can LH, Basci A, Ok E (2009) The benefit of salt restriction in the treatment of end-stage renal disease by haemodialysis. Nephrol Dial Transplant 24:956-962.
- Mc Causland FR, Waikar SS, Brunelli SM (2012) Increased dietary sodium is independently associated with greater mortality among prevalent hemodialysis patients. Kidney Int 82:204-211.
- Maduell F, Navarro V (2000) Dietary salt intake and blood pressure control in haemodialysis patients. Nephrol Dial Transplant 15:2063.
- Shaldon S. (2002) Dietary salt restriction and drug-free treatment of hypertension in ESRD patients: a largely abandoned therapy. Nephrol Dial Transplant. 17(7):1163-5.
- McCausland FR, Qaikar SS, Brunelli SM (2013) The relevance of dietary sodium in hemodialysis. Nephrol Dial Transplant 28: 797–802

How to reduce salt intake

- Kobayashi S, Amano H, Terawaki H (2025) Impaired saltiness perception contributes to higher sodium intake among patients with chronic kidney disease: a cross-sectional two-center study. J Ren Nutr. 35:103-109
- MacGregor GA, Sever PS (1996) Salt--overwhelming evidence but still no action: can a consensus be reached with the food industry? CASH (Consensus Action on Salt and Hypertension). BMJ. 312(7041):1287-9
- Blais CA, Pangborn RM, Borhani NO, Ferrell MF, Prineas RJ, Laing B (1986) Effect of dietary sodium restriction on taste responses to sodium chloride: a longitudinal study. Am J Clin Nutr. 44(2):232-43
- Rauber F., Campagnolo,P., Hoffman D., Vitolo M (2015) Consumption of ultra-processed food products and its effects on children’s lipid profiles: a longitudinal study. Nutrition, Metabolism and Cardiovascular Diseases, 25(1):116-122.
- Humalda JK, Klaassen G, de Vries H, Meuleman Y, Verschuur LC, Straathof EJM, Laverman GD, Bos WJW, van der Boog PJM, Vermeulen KM, Blanson Henkemans OA, Otten W, de Borst MH, van Dijk S, Navis GJ; SUBLIME Investigators (2020) A Self-management Approach for Dietary Sodium Restriction in Patients With CKD: A Randomized Controlled Trial. Am J Kidney Dis. 75(6):847-856
- Quader ZS, Gillespie C, Sliwa SA, Ahuja JK, Burdg JP, Moshfegh A, Pehrsson PR, Gunn JP, Mugavero K, Cogswell ME (2017) Sodium Intake among US School-Aged Children: National Health and Nutrition Examination Survey, 2011-2012. J Acad Nutr Diet. 117(1):39-47
- van Rossum, C.T.M., et al., Dutch National Food Consumption Survey 2007-2010 : Diet of children and adults aged 7 to 69 years, in Nederlandse voedselconsumptiepeiling 2007-2010 : Voeding van kinderen en volwassenen van 7 tot 69 jaar. 2011, Rijksinstituut voor Volksgezondheid en Milieu RIVM.
- WHO 2014. ISBN 978 92 4 150669 4 Salt reduction and iodine fortification strategies in public health: report of a joint technical meeting convened by the World Health Organization and The George Institute for Global Health in collaboration with the International Council for the Control of Iodine Deficiency Disorders Global Network, Sydney, Australia, March 2013.
- Verkaik-Kloosterman J, Buurma-Rethans EJM, Dekkers ALM, van Rossum CTM (2017) Decreased, but still sufficient, iodine intake of children and adults in the Netherlands. Br J Nutr. 117(7):1020-1031
- Jakobsen LS, Nielsen JO, Paulsen SE, Outzen M, Linneberg A, Møllehave LT, Christensen T, Ravn-Haren G (2022) Risk-Benefit Assessment of an Increase in the Iodine Fortification Level of Foods in Denmark-A Pilot Study. Foods 11(9):1281
- Bath SC, Verkaik-Kloosterman J, Sabatier M, Ter Borg S, Eilander A, Hora K, Aksoy B, Hristozova N, van Lieshout L, Tanju Besler H, Lazarus JH (2022) A systematic review of iodine intake in children, adults, and pregnant women in Europe-comparison against dietary recommendations and evaluation of dietary iodine sources. Nutr Rev. 80(11):2154-2177
- Olivieri A, Trimarchi F, Vitti P (2020) Global iodine nutrition 2020: Italy is an iodine sufficient country. J Endocrinol Invest. 43(11):1671-1672
